# Bile acid derivatives as novel co-adsorbents for enhanced performance of blue dye-sensitized solar cells

**DOI:** 10.1038/s42004-025-01433-1

**Published:** 2025-03-10

**Authors:** Kezia Sasitharan, Allan J. Mora Abarca, Fabio Cucinotta, Leslie W. Pineda, Victor Hugo Soto Tellini, Marina Freitag

**Affiliations:** 1 School of Natural and Environmental Science, Newcastle University, Newcastle Upon Tyne, UK; 2 Centro de Investigación en Electroquímica y Energiá Química (CELEQ), Universidad de Costa Rica, San Jos, Costa Rica

**Keywords:** Energy, Physical chemistry, Devices for energy harvesting, Solar cells

## Abstract

Diketopyrrolopyrrole-based blue dyes in dye-sensitized solar cells (DSCs) exhibit promise for building-integrated photovoltaics, but their efficiency is compromised by dye aggregation-induced charge recombination. Novel bile acid derivative co-adsorbents featuring bulky hydrophobic substituents at the 3-*β* position were synthesized to address this challenge. These molecules, designed to modulate intermolecular electronic interactions, effectively altered the TiO_2_ surface coverage dynamics, as evidenced by UV-Vis spectroscopy and dye-loading kinetics. Systematic variation of hydrophilic substituents revealed structure-function relationships in dye separation efficacy. Devices incorporating these co-adsorbers achieved power conversion efficiencies (PCE) of 7.6%, surpassing reference devices (5.2%) and those using conventional chenodeoxycholic acid co-adsorbers (6.4%). The optimized devices exhibited a 30% increase in short-circuit current density, 30 mV enhancement in open-circuit voltage, and 60% peak external quantum efficiency at 550 nm. Time-resolved photoluminescence spectroscopy confirmed suppressed non-radiative recombination, while transient absorption spectroscopy revealed accelerated electron injection rates from 6.4 ps to 4.6 ps. Electrochemical impedance spectroscopy elucidated the mechanism of reduced interfacial recombination. These findings present a molecular engineering strategy for mitigating lateral charge transfer in planar dye systems, advancing semi-transparent hybrid photovoltaics.

## Abstract (web summary)

Diketopyrrolopyrrole-based blue dyes in dye-sensitized solar cells (DSCs) exhibit promise for building-integrated photovoltaics, but their efficiency is compromised by dye aggregation-induced charge recombination. Here, to address this challenge, the authors synthesize bile acid derivative co-adsorbents featuring bulky hydrophobic substituents at the 3-*β* position and demonstrate that the corresponding devices achieve power conversion efficiencies of 7.6%, surpassing those with conventional co-adsorbers

## Introduction

The global pursuit of sustainable energy solutions has led to significant advancements in photovoltaic technologies. As the world increasingly focuses on reducing carbon emissions and enhancing energy efficiency in urban environments, the integration of solar energy harvesting into building materials has become a key area of research and development. Semi-transparent solar cells have emerged as a promising technology for building-integrated photovoltaics (BIPV)^1^, offering a dual functionality of energy generation and aesthetic appeal in modern architecture^2^. Among the various thin-film technologies, dye-sensitized solar cells (DSCs) stand out for their potential in creating colorful, semi-transparent photovoltaic windows. However, the challenge lies in developing DSCs that can efficiently harvest light in the near-UV and near-IR regions of the solar spectrum while maintaining transparency in the visible range^3^. This delicate balance between power conversion efficiency and aesthetic qualities is crucial for the widespread adoption of DSCs in BIPV applications. In this context, diketopyrrolopyrrole (DPP) based blue dyes have shown promise, offering both efficient light harvesting in the red and near-IR parts of the spectrum and an appealing blue color. These planar dyes face a critical challenge: aggregation on the photoanode surface, which significantly impedes their power conversion efficiency. This study addresses this issue by exploring novel bile acid derivatives as co-adsorbents, designed to mitigate dye aggregation and enhance the performance of DPP-based blue dye solar cells.

Amongst blue-colored sensitizers, DPP based dyes are notable for their high performance, offering a good balance between aesthetic properties and efficient harvesting of the red and near-IR parts of the solar spectrum^4,5^. With a push-pull dye structure composed of a donor-*π*-conjugate bridge-acceptor motif, DPP dyes assume a planar configuration when adsorbed onto a solid surface like TiO_2_^6^. DPP-based blue dyes incur a large transient dipole moment upon photoexcitation and therefore experience significant lateral electronic interactions once adsorbed on a solid surface^7^. This is due to the acceptor being chemically linked to the donor via a conjugate bridge making intermolecular charge transfer possible when the dye molecules stack together and form supramolecular aggregates^8^. The intermolecular electronic interactions between dye molecules in such aggregates when adsorbed on a mesoporous solid oxide surface such as TiO_2_ are crucial for electron transfer processes in DSCs. These interactions can facilitate various processes such as reductive quenching upon dye aggregate formation leading to the loss of the photoexcited state^9^. This in turn affects the kinetics and efficiency of interfacial charge transfer upon photoexcitation leading to an overall decrease in the power conversion efficiency^10^.

Hence, optimal and ordered coverage of dye molecules is essential on the mesoporous layer, in addition to good light absorption, injection, and aesthetic characteristics to enhance power conversion efficiencies^11^. Prior research has shown that judicious engineering of the sensitizer molecular structure such as incorporation of 2,4-bishexyloxybiphenyl steric bulk on the DPP donor moiety can significantly improve the open circuit voltage and power conversion efficiencies^12^. Additionally, modulating the anchor and chromophore of the DPP moiety has been reported to help balance aggregation, red light response, and electron-withdrawing strength^13^, whilst co-sensitization has been shown to enhance the photocurrent due to a wider light absorption range^14^. In pursuit of improved performance, anti-aggregation agents (also referred to as co-adsorbents) are widely used to enable better coverage of the dye molecules. So far, all literature reports using DPP-based dyes are usually adsorbed from dye containing a large excess of the co-adsorbents to reduce dye aggregation on the surface^5^. Widely employed co-adsorbers in DSCs are bile acids and salts (predominantly cholic acid derivatives) which are biological surfactants with a non-conventional amphiphilic nature^15^. They are typically characterized by their convex hydrophobic side (called the -*β*-side) and a concave hydrophilic side (-*α*-) combined with a negatively charged end of a side chain. Owing to their unique distribution of the polar and apolar regions and structural rigidity, bile salt molecules and their derivatives can form micellar aggregates^16^. Chenodeoxycholic acid (CDCA) is the widely employed bile acid co-adsorbent known to minimize dye/dye interactions. Fine-tuning the hydrophilic-hydrophobic features of the CDCA molecules has shown some unique supramolecular features^17,18^. Previously, attaching sterically demanding amide groups instead of the typical terminal hydroxyl groups in CDCA was found to result in comparable current densities albeit slightly lower PCEs^19^. Building on these findings, in this work we establish tethering of bulky groups to the bile acid derivatives as a viable route for DPP-like sensitizers where a large excess of co-adsorbents is typically essential.

**Fig. 1:**
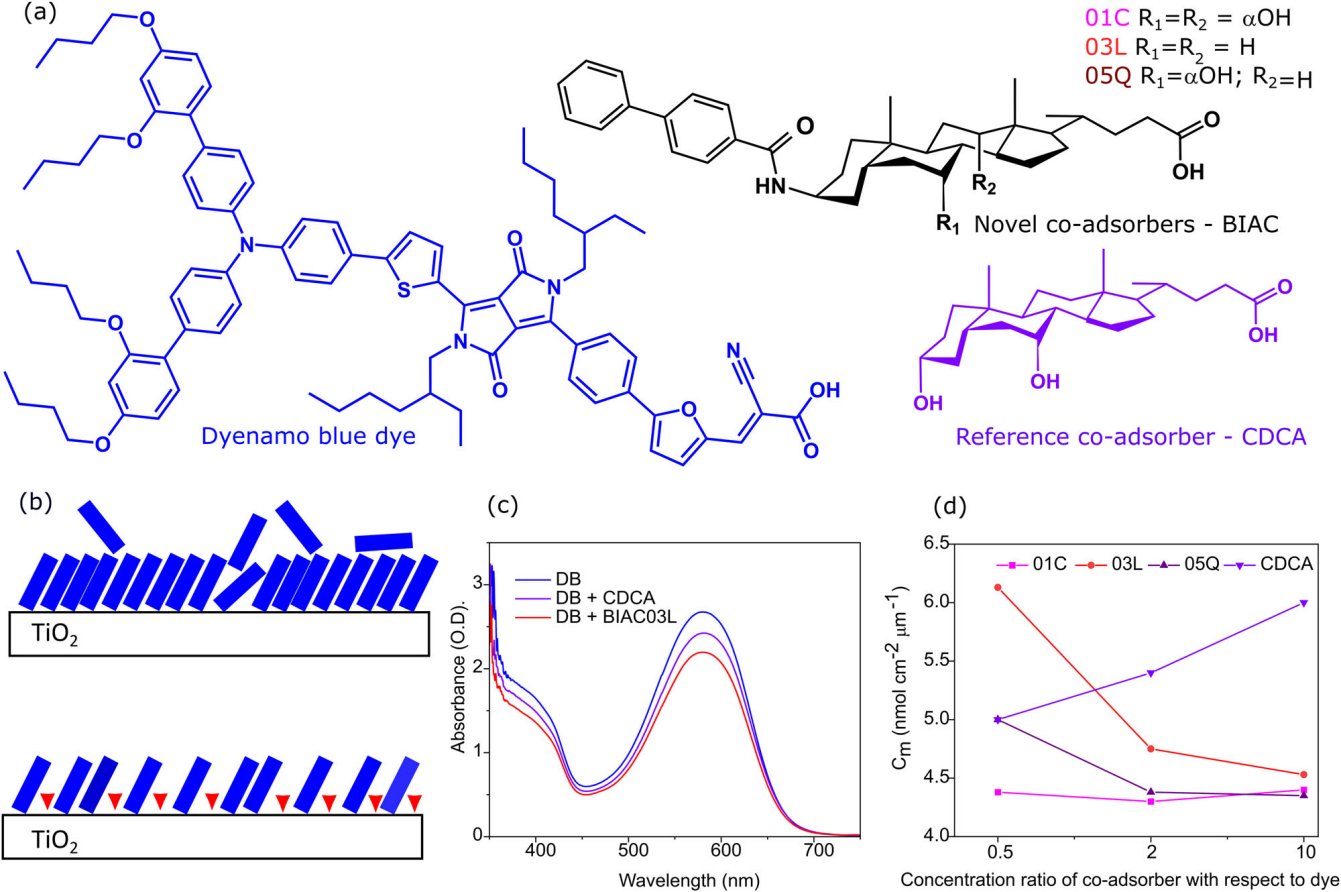
Molecular structures and the effect of bulky co-adsorbers on dye uptake. **a** Molecular structures of Dyenamo blue, the novel co-adsorbers BIAC01C, BIAC03L and BIAC05Q, and the reference co-adsorber CDCA. **b** Schematic representation of the adsorption process of the dye in the absence and presence of the bulky co-adsorbers (**c**) UV-Vis absorption spectra of the pristine dye and the dye with the co-adsorbers on mesoporous titania films. **d** Plot of the loading amount of the dye on the titania films in the presence of the co-adsorbers as a function of increasing concentration.

Here-in we explore the role of attaching bulky hydrophobic groups Fig. 1a at the 3-*β*-position towards restraining dye aggregation on the mesoporous surface. We have also systematically varied the number of hydrophilic substituents and evaluated how this modification could favor the separation between the dye molecules. Primarily, we evaluate the effect of the steric hindrance features of the substituents on the photovoltaic performances of the devices. Through expanding the hydrophobic 3-*β*-region the new co-adsorbers favor separation between the dye molecules leading to substantial improvements to the photocurrent and power conversion efficiency. Importantly we find that the impeded dye aggregation leads to improved charge carrier density which allows for the first time to attain a PCE exceeding 7.5% with a dyenamo blue dye-based DSC. We hypothesize that using co-adsorbers with bulky substituents slows down the uptake of the dye molecules enabling improved ordering on the mesoporous surface (Fig. 1b).

## Results

### Effect of bulky co-adsorbers on dye coverage on mesoporous titania

We introduce bulky substituents in the 3-*β*-position of the co-adsorbate molecules to form BIAC (01C, 03L and 05Q) structures (Fig. 1a) that serve to counteract aggregation and recombination at the mesoporous surface (Fig. 1b). The synthetic routes of BIAC01C, BIAC03L and BIAC05Q are depicted in supplementary Scheme1 and all the intermediates and target compounds have been characterized by 1H nuclear magnetic resonance (NMR), 13C NMR and high-resolution mass spectrometry (Supplementary Figs. 8–37). As shown in Fig. 1c, the spectral absorption profiles of the dye are not affected by the co-adsorbers, retaining the intense absorption peak at 580 nm and a shoulder around 400 nm.

It is worth noting that the intensity of the absorption spectra is lower for the films prepared with the co-adsorbers as compared to the pristine dye bath. The absorption intensity is also lower for the films with the novel co-adsorbers as compared to the films with the reference CDCA co-adsorber. This shows that the 3*β* substituents have a significant effect on the adsorption of the dye molecules. The decrease in absorbance is directly related to a decreased amount of the adsorbed dye^15^. For further analysis, we measured the kinetics of the dye uptake on the mesoporous titania in the presence of the bulky co-adsorbers. For this, we measured the dependence of the concentration of the co-adsorber in the dye bath of the number of moles of adsorbed sensitizer per unit geometric area and unit film thickness C_m_ for each co-adsorber in comparison with the reference S31. Figure 1d shows that in the presence of the reference CDCA co-adsorber, the C_m_ values for Dyenamo blue increase rapidly as the concentration of the co-adsorber increases. This indicates that the reference CDCA co-adsorber inhibits the desorption of the dye resulting in substantially more dye accumulating on the titania surface indicating a denser molecular packing. However, for the novel co-adsorbers developed by us, we observe that the dye uptake was reduced as the concentration of the co-adsorber increased from 0.5 to 2 fold, and then stabilized upon a further 10-fold increase of the concentration of the co-adsorber. (Fig. 1d). This proves that replacing the hydroxyl group at the 3-*β*- position of CDCA with a bulky group retards the dye adsorption process by steric hindrance and separates the dye molecules more effectively.

### Effect of co-adsorbers on solar cell efficiency

**Fig. 2:**
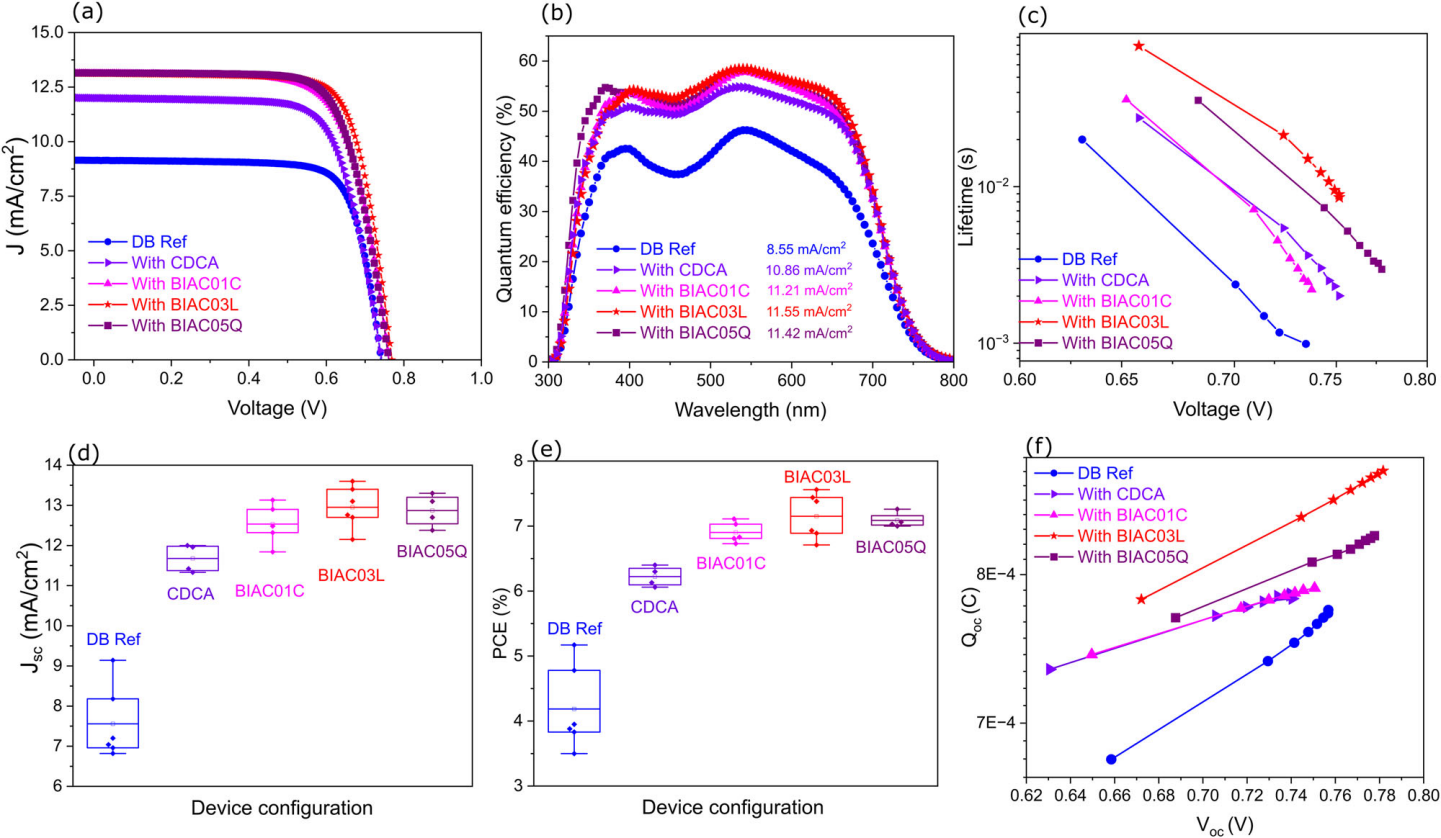
Effects of the co-adsorbers on the device performance. **a** Current-voltage curves measured under AM1.5G simulated solar illumination. **b** EQE spectra of the devices with the corresponding integrated photocurrents listed in the legend. **c** Electron lifetime of the solar cells with the different co-adsorbers. **d** Box plot showing the distribution of current densities of the devices based on the co-adsorbers. **e** Box plot showing the distribution of power conversion efficiencies of the devices. **f** Extracted charge in the solar cells as a function of open circuit potential.

We fabricated DSCs with TiO_2_ electrodes sensitized with DB with and without co-adsorbers. Cobalt tris(bipyridine) was used as the redox mediator in the electrolyte. This one-electron redox couple is highly suited for DPP-based sensitizers offering sufficient driving force for electron injection from the excited state into the conduction band of the mesoporous titania. Detailed device fabrication and characterization are described in the methods section. Plots of the current-voltage curves of the optimal performing devices measured under an air mass of 1.5 global (AM 1.5G) simulated solar illumination at 100 mW cm^−2^ are shown in Fig. 2a. The corresponding PV parameters are listed in Table 1 and the statistical analysis is tabulated in supporting Table S7. Figure 2b shows the incident photon-to-electron conversion (IPCE) efficiency spectra of the optimal devices. Without any co-adsorbers, the best DB dye-based device achieved a PCE of 5.17% with a *J*_SC_ of 9.14 mA cm^−2^, a *V*_OC_ of 0.74V, and a fill factor (FF) of 0.76. Four individual devices built with each co-adsorber were analyzed statistically and the reproducibility of the device metrics is reported in Fig. 2d–e. All the co-adsorbers regardless of the concentration ratio give better efficiencies compared to the devices without co-adsorbers (Supplementary Fig. S32, S33, S34). Notably, all the bulky co-adsorbers give significantly greater efficiencies than the reference CDCA co-adsorbere. These findings indicate that bulky groups at the R3 terminal position of the co-adsorbates play a role in improving the performance of the DSC devices. The integrated short circuit current density from the IPCE correlates well with the statistical analysis from our current-voltage data. While all these results show that the bulky substituents favor higher performance of the dye, we cannot rule out the influence of the presence or absence of the hydroxyl substituents in the backbone of the bile acids as this has previously been shown to strongly favor supramolecular interactions. The integrated photocurrent density calculated from the IPCE spectra (11.55 mA cm^−2^) shows a ~10% difference from the *J*_SC_ measured under AM1.5G illumination (12.96 mA cm^−2^). This discrepancy arises from the different measurement conditions—IPCE is measured under monochromatic illumination while J–V curves are obtained under broadband AM1.5G illumination.

**Table 1:** Photovoltaic performance of the Dyenamo blue DSC devices based on the co-adsorbers CDCA, BIAC01C, BIAC03L, BIAC05Q under AM1.5G sunlight

Dye:Co-sensitizer	Jsc (mA/cm^2^)	Voc (V)	FF	PCE (%)
DB	9.14	0.74	0.76	5.17
DB:CDCA	12.00	0.74	0.72	6.40
DB:BIAC01C	13.13	0.76	0.71	7.11
DB:BIAC03L	13.10	0.77	0.75	7.56
DB:BIAC05Q	13.10	0.76	0.73	7.26

Note that a high FF was achieved in the reference device without co-adsorbers. The optimal device with the reference co-adsorber CDCA had a lower FF of 0.72, a comparable V_oc_ of 0.74V, a higher *J*_SC_ of 12 mA/cm^2^, and a higher PCE of 6.4%. It is noteworthy that all the devices with the novel co-adsorbers BIAC demonstrate a higher PCE as compared to both DB reference device and the CDCA reference co-adsorber. Solar cell characterization of the DSCs with different dye loadings is shown in ESI Fig. S32, S34. Upon increasing the co-adsorber concentration, the *V*_OC_, *J*_SC_, and FF of the device increase. We anticipate that the charge collection efficiency improves with the increase in co-adsorber concentration due to a more ordered coverage of the mesoporous titania layer.

Furthermore, we have evaluated the recombination kinetics within the cells using transient photovoltage analysis. The electron recombination lifetimes of the dye under different co-adsorbers are shown in Fig. 2c. Here, the lifetime refers to the time that the injected electron can spend in the porous TiO_2_ before recombining with either the dye cation or the acceptor species in the cobalt electrolyte. The recombination lifetimes show a clear trend from DB < CDCA < BIAC01C < BIAC05Q < BIAC03L. This correlates with the trend in *V*_OC_ values for the different cell types, indicating that the DB reference cell without co-adsorbers has the highest rate of electron recombination leading to the shortest electron lifetime. The cells with the bulky co-adsorbers were found to have longer electron lifetimes as compared to the standard CDCA co-adsorber. The longer lifetime indicates more effective suppression of the back reaction of the injected electron with the cobalt electrolyte due to the better-ordered dye arrangement on the mesoporous layer enabled by the bulky amide substituents. Charge extraction measurements were performed on the DSC devices under open circuit conditions. The extracted charge under open circuit conditions Q_OC_ Fig. 2f was very similar for devices regardless of the co-adsorber used. This suggests that there are no significant changes in the conduction band edge position. In the absence of any co-adsorber, the reference cell demonstrates deviating behavior showing the charge collection efficiency is low in the absence of co-adsorbers.

### Effect of co-adsorber in co-sensitized solar cells

**Fig. 3:**
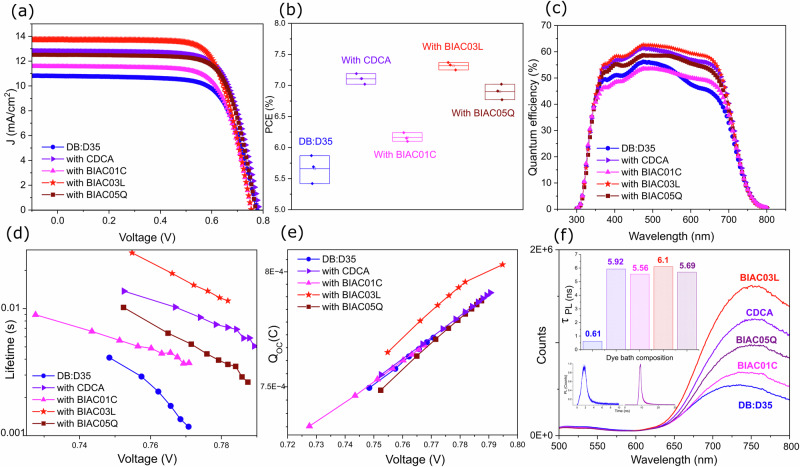
Effect of the co-adsorbers on the device performance of co-sensitized cells. **a** Current density-voltage (J–V) curves measured under AM1.5G simulated solar illumination. **b** Box plot showing the distribution of the power conversion efficiencies for the co-sensitized devices. **c** EQE spectra of the co-sensitized devices. **d** Electron lifetime of the solar cells with the different co-adsorbers. **e** Extracted charge in the solar cells as a function of open circuit potential. **f** Photoluminescence spectra of the films on zirconium dioxide stained in the dye bath with co-adsorbers for 12 h. The inset shows the time-resolved PL spectra of the co-sensitized films. The photoluminescence lifetime at 12 h staining of the DB+ D35 co-sensitized ZrO_2_ films with and without the various co-adsorbers are shown in the inset histogram plot.

To further evaluate the effect of the co-adsorbers we chose the commonly used co-sensitizer for blue dyes—D35 with DB. As shown in Fig. 3a the co-sensitized DB+ D35-based DSC retains the high performance. The BIAC03L co-adsorber continues to yield the highest-performing cells. The optimized device exhibits an enhanced PCE of 7.37% under simulated standard AM 1.5G, 100 mW cm^−2^ with a current density of 13.73 mA/cm^2^, a *V*_OC_ of 0.77, and FF of 0.71. The higher *J*_SC_ found under solar irradiation in the systems with the co-adsorber correlates with improved IPCE values. The IPCE values of the D35+ DB systems in the presence of the co-adsorbers show a remarkable increase within the wavelengths ranging from 500 to 600 nm (DB absorption region) indicating that the co-adsorbers can suppress the aggregation of DB on the TiO_2_ films during the dye uptake process when in a co-sensitization environment as well. Supplementary Table S8 details the photovoltaic performance of the DB:D35 co-sensitized DSC devices based on the co-adsorbers CDCA, BIAC01C, BIAC03L, BIAC05Q under AM1.5G sunlight.

Complete devices were used to measure electron lifetimes by means of voltage response to small modulations in the light. The electron lifetimes correspond to the time for a recombination of the electron in the mesoporous TiO_2_ to the oxidized component in the electrolyte ${{\rm{Co}}}{{({{\rm{bpy}}})}_{3}}^{3+}$, and to the oxidized dye if the dye regeneration is slow. The recombination lifetimes show a trend of DB < BIAC01C < BIAC05Q < CDCA < BIAC03L—correlating with the *V*_OC_ values from the devices. The longer lifetime indicates more effective suppression of the back reaction of the injected electron with the cobalt electrolyte. This is also shown in the increased photocurrent in the devices. We further performed time-resolved photoluminescence (TRPL) spectroscopy measurements of co-sensitized zirconium dioxide (ZrO_2_) at 12 h staining with the different co-adsorbers. This was done to better understand the electron injection process of the dye loaded onto the metal oxide surface in the presence of the co-adsorbers. Zirconium dioxide is a high band gap material and is therefore used in this case as a non-injecting substrate whilst bearing a closer resemblance in its mesoporous structure to titania. The photoluminescence spectra of the films on zirconium dioxide are displayed in Fig. 3f. Significant quenching is observed in the absence of the co-adsorbers, with BIAC03L samples displaying the least quenching. Since the energy level of the conduction band of zirconia is too high, electrons cannot be injected from the excited dye into the metal-oxide layer. Therefore, the lateral intermolecular interactions are responsible for the observed quenching rather than interfacial electron transfer. Hence, the reduced quenching exhibited by the sample containing our best-performing co-adsorber—BIAC03L signifies improved inhibition of the lateral intermolecular interactions between the dye molecules which in turn leads to superior device performance. The PL decay profile was fitted with a monoexponential decay and the observed photoluminescence lifetimes are shown as the inset in Fig. 3f. Since the TRPL probing wavelength is 760 nm in which DB mostly emits and D35 very little, the calculated PL lifetimes can be mostly attributed to DB. Figure 3f inset shows that the calculated photoluminescence lifetime of the samples with the co-adsorbers was prolonged by 89% as compared to the pristine dye (Supporting S9). The prolonged *τ*_PL_ values indicate that the non-radiative inter-molecular recombination was suppressed in the packed dye layer in the presence of the bulky co-adsorbers. This further substantiates our claim that staining the mesoporous layer in the presence of the bulky co-adsorbers leads to a more ordered dye packing and reduced lateral intermolecular interactions.

### Interfacial charge transfer characteristics

**Fig. 4:**
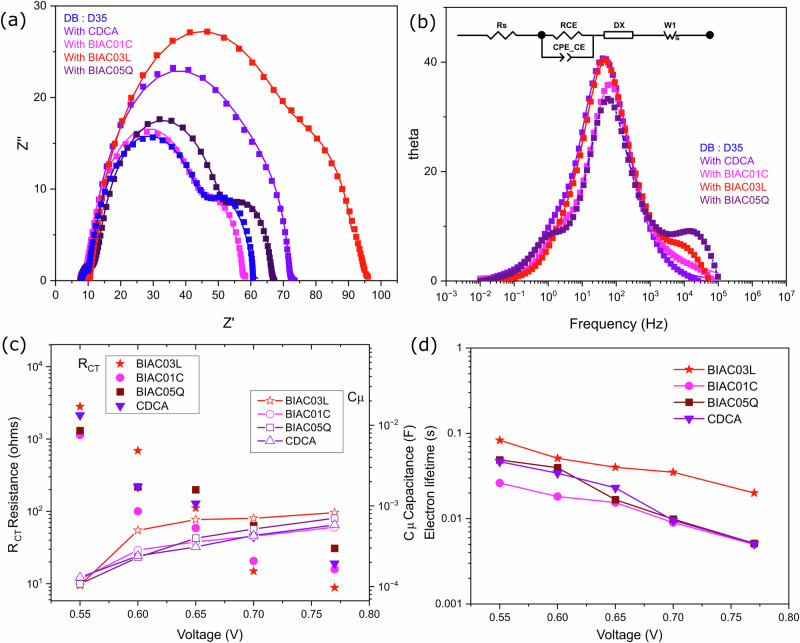
Effects of the co-adsorbers on the interfacial charge transfer characteristics evaluated by electrochemical impedance spectroscopy. **a** Nyquist plots. **b** Bode plots. **c** Plots of charge transfer resistance and chemical capacitance as a function of voltage for DSCs based on the different co-adsorbers. **d** Electron recombination lifetime as a function of voltage for the cells with different co-adsorbers.

We carried out electrochemical impedance spectroscopy (EIS) to further investigate the interfacial charge transfer kinetics. EIS experiments were performed on complete devices under white light LED illumination for an in-depth analysis of the energetics and kinetic features of the electron processes in the devices. We evaluated the obtained data with the ZView software, fitted using the transmission line model, presented in Fig. 4b^20^. The given data successfully models the charge transfer and chemical capacitance at the interface of TiO_2 _dye/electrolyte and PEDOT counter electrode/electrolyte under operational conditions (*V*_OC_). Combination of charge transfer resistance (R_CT_) and chemical capacitance gives rise to the semicircles in the complex plane as observed in the Nyquist plots shown in Fig. 4a. The first semicircle at high frequency corresponds to the PEDOT counter electrode, the mid semicircle corresponds to the charge transfer resistance at the interface of the dye/TiO_2_ combined with the chemical capacitance of electrons in TiO_2_ and the third semicircle corresponds to a finite Warburg impedance element. Figure 4a shows the effect of the different co-adsorbers on the DB:D35 co-sensitized system. The intermediate frequency range corresponding to the electron recombination resistance follows the order BIAC03L> CDCA> BIAC05Q> BIAC01C. One possible reason for this is that the dye molecules adsorbed on the semiconductor surface control the rate of injected electrons. The enhanced bulkiness of the BIAC03L co-adsorber leads to a more compact monolayer formation which decreases the area that is active for recombination preventing the recombination of conduction band electrons back to the electrolyte^21^. While in principle, this holds true for the BIAC01C and O5Q as well, they are different from the high-performing BIAC03L in terms of additional hydroxyl groups on the *α* side of the bile acid. These hydroxyl groups can favor strong supramolecular interactions dictating how they self-assemble and reorganize themselves on a mesoporous solid-state support such as titania. We anticipate that in a co-sensitization scenario, these additional supramolecular effects introduced by the hydroxyl groups do not deactivate the annihilation of the excited dye molecules as efficiently as BIAC03L leading to decrease in electron injection efficiencies. Therefore, the higher the charge recombination resistance in the Nyquist plots, the higher the photovoltage obtained from the devices due to the slower charge recombination of electrons—consistent with our observations in the device performances. The chemical capacitance values of BIAC03L exceed the other co-adsorbers further confirming effective passivation of the TiO_2_ surface by the dye monolayer to reduce recombination between the electrons in TiO_2_ and electrolyte. These results indicate that the solar cells with BIAC03L co-adsorbers have the slowest interfacial recombination amongst all. The electron lifetime derived from the EIS measurements (the product of recombination resistance and chemical capacitance) follows the same trend as that from the transient photovoltage analysis in the previous section.

### Effect of co-adsorber on the lateral intermolecular electronic interactions

With the organic dyes being tightly packed on the mesoporous metal-oxide surface, it is important to investigate the kinetics of the interfacial dynamics as well as the lateral interactions arising from the short intermolecular distances to get a comprehensive understanding of the dye-co-adsorber interactions. We examine the detailed dynamics of the dye in the presence of the reference co-adsorber and the bulky co-adsorber introduced in this work by comparing their characteristics both in solution state as well as adsorbed on a redox inactive zirconia substrate.

#### Solution studies

**Fig. 5:**
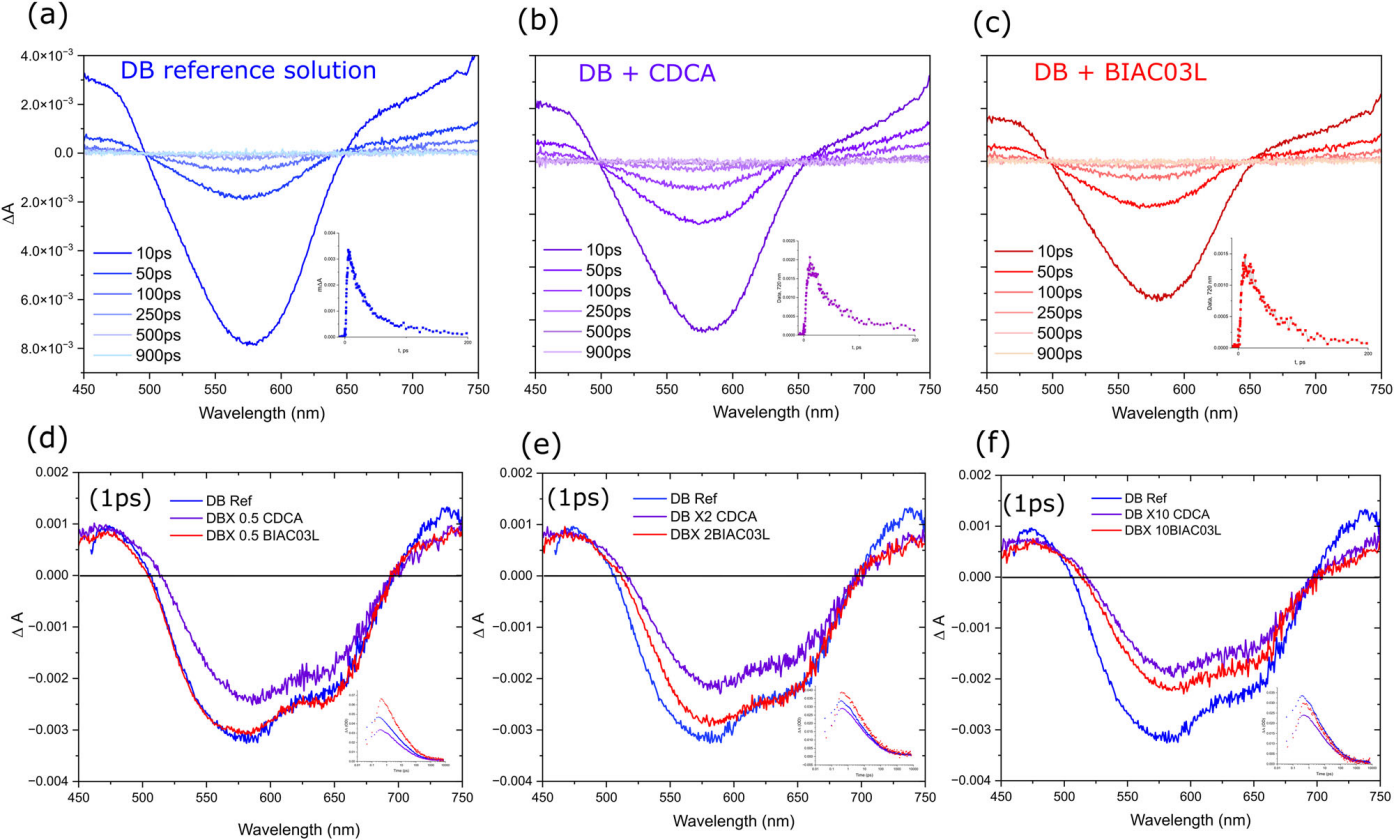
Transient absorption spectra to evaluate the effect of co-adsorbers on the lateral intermolecular electronic interactions. **a**–**c** Solution state TA spectra of dyenamo blue, dyenamo blue with CDCA and dyenamo blue with BIAC03L co-adsorber in a 4-tert-butanol/acetonitrile solution (1:1 V/V) at different optical delays (**d**–**f**). TA spectra of dyenamo blue films on zirconium dioxide at optical delay of 1 ps, plotted to compare against different concentrations of the co-adsorbers CDCA and BIAC03L.

The *Δ*A spectra for the dye and dye+co-adsorber solutions were recorded to identify the characteristic bands and transition energies pertaining to S_1_ to S_n_ excited state absorption. Figure 5a–c shows the fs-TAS spectra at selected delay times for DB solution, DB solution with CDCA co-adsorber, and BIAC03L co-adsorber respectively in the UV-Vis to NIR region (450–750 nm). Concentration of 7 μM for DB dye with (×10 times the co-adsorber) was used to observe the intermolecular behaviors in the solution phase. The delay time only affects the amplitude and not the shape of the *Δ*A spectra. All samples show ground-state bleaching with peaks at 580 nm which overlap with the ground-state absorption spectra when inverted indicating an S_0_ to S_1_ transition and the excited state absorptions are superimposed on either side. The solutions with the co-adsorbers—CDCA and BIAC03L exhibit identical features and band shapes and we expect the same in the films as well. The excited state lifetimes obtained from the average multiexponential fits to the transients of the dye provide rising component of 2.1 ps followed by a 23.3 ps decay component in good agreement with previous reports^8,22^. In the solutions with co-adsorbers, the average multiexponential fits to the transients of DB+CDCA and DB+BIAC03L provide rising components of 3.26 and 2.8 ps, respectively followed by 40.2 and 38 ps decay components. The decrease in the fitted time components of the pristine dye solution is attributed to new deactivation channels induced by intramolecular charge transfer characteristics of the excited state which leads to the accelerated decay process.

#### Dye and co-adsorbers grafted onto zirconium dioxide films

In this section, we investigate the kinetic traces of the interfacial dynamics and the lateral interactions arising from the short intermolecular distance when adsorbed onto a mesoporous metal oxide surface. The photo-induced charge transfer processes include electron injection from dye molecules into semiconductor acceptor states and dye regeneration by the redox electrolyte. Previous studies employ alumina or zirconia as inert reference materials due to the high energy of its conduction band which prevents electron injection from excited dyes^8,22,23^ In this work, zirconia has been used to study the excited state dynamics of the dye molecules in the presence of the co-adsorbers when bound to a metal oxide surface and to calculate the electron injection yields into the mesoporous non-inert substrate (titania) by comparison of the excited state lifetimes. We anticipate that a charge separation process might occur between dye molecules when they are adsorbed to an inert metal oxide surface and that charge transfer can be inhibited by controlling the packing of molecules on the surface through use of our bulky co-adsorbers that separate the dye. Figure 5d–f shows selected TA spectra from the investigation of the femtosecond dynamics of dye DB adsorbed onto zirconium dioxide on its own and in combination with the co-adsorbers CDCA and BIAC03L. The positive band rising at 700 nm in the DB films seems sensitive to the presence and concentration of the co-adsorbers as noted by the quenching in the intensity in the films with CDCA and BIAC03L. In the pristine dye films (without co-adsorbers), we expect dye aggregation. Therefore the intermolecular distances between dye molecules will be shortened, accelerating the lateral intermolecular interactions. The rising components being faster in the films without co-adsorbers confirms that lateral intermolecular interactions occur more effectively when the distance between the molecules is shorter. The kinetics are strongly affected in the presence of both CDCA and BIACO3L as evidenced by the change in both the rise and decay components with increase in concentration of the co-adsorbers. The fast-rising component of 3.4 ps in the TA kinetics of the pristine dye samples suggests that the driving force for the occurrence of these lateral intermolecular interactions is larger when the films are prepared with the dye alone than when in the presence of a co-adsorber. Compared to the rising component of 7 ps reported for the dye-sensitized zirconia films prepared by adding the co-adsorbents, the rising component of the corresponding titania films prepared under identical conditions appear to be faster (4.6 ps) and average decay component for the deactivation of the charge separation state formed from the intermolecular interactions is found to be 90 ps. This is much faster than the average component observed in the zirconia films (245 ps for CDCA and 188.9 ps for BIAC03L).

## Discussion

Dye aggregation on the photoanode surface of DSCs can significantly impede their power conversion efficiency. This effect is pronounced in diketopyrrolopyrrole-based dyes such as Dyenamo Blue where due to the large transient dipole moment in the structure, lateral charge transfer between neighboring dye molecules occurs upon photo-excitation. This affects the efficiency of the electron injection leading to lower PCE. To address this issue, in this work, we investigated the use of bile acid derivatives as co-adsorbents to mitigate dye aggregation and enhance DSC performance. A series of bile acid derivatives were synthesized, systematically modifying the 3-*β* position with bulky hydrophobic groups and varying the number of hydrophilic substituents in the backbone. We demonstrate that introducing bulky co-adsorbers is a good strategy to maintain good adsorption-injection characteristics of dyenamo blue dyes on mesoporous surfaces. Results revealed that bulky hydrophobic groups at the 3-*β* position retards the uptake of dye molecules and effectively inhibits dye aggregation, leading to a better packing density of the dye molecules on the TiO_2_ surface. Notably, in planar dyes such as dyenamo blue, all the co-adsorbents with the bulky hydrophobic substituents and additional hydrophilic substituents showed a substantial improvement with a power conversion efficiency of >7% as compared to 5.17% in the reference device without co-adsorbers and 6.4% in the devices with a conventional co-adsorber that does not comprise the bulky hydrophobic substituent. Interestingly, the best-performing device at 7.56% PCE, with a 30% increase in short-circuit current density (*J*_SC_) and a 30 mV increase in open-circuit voltage (*V*_OC_) was observed with the co-adsorber that had an expanded hydrophobic 3*β* region but no hydroxyl substituents on the hydrophilic side. External quantum efficiency (EQE) measurements showed a maximum value of 60% at 550 nm for the optimized device, compared to 45% for the reference device accompanied by a near 30% increase in integrated photocurrents. This is supported by longer electron lifetime revealed from the transient photovoltage and charge collection measurements indicating a more effective suppression of the back reaction of the injected electron with the cobalt electrolyte. In a co-sensitized dye solar cell, the optimized co-adsorber continues to be the best-performing system with a PCE of 7.37% as compared to 5.87% in the reference device accompanied by an increase in the current density and a 30 mV increase in the open circuit voltage. In comparison, the devices with CDCA co-adsorber that do not include the bulky amide substituents at 3-*β* position showed a PCE of 7.19%. It is noteworthy that the current densities reach 13.73 mA/cm^2^ in the BIAC03L devices as compared to 10.8 mA/cm^2^ and 12.82 mA/cm^2^ in the reference devices with no co-adsorber and the reference devices with the conventional co-adsorber, respectively. This increase in photocurrent is also corroborated by the increased electron lifetime obtained by both transient photovoltage and impedance techniques re-iterating the role played by the bulky co-adsorbers in suppressed dye aggregation, leading to suppressed recombinations. We hypothesized that the improved photocurrent arises from the suppression of the deactivation of the excited states via quenching processes between dye molecules. To confirm this we conducted a series of ultrafast spectroscopic evaluations. Time-resolved photoluminescence measurements revealed a prolonged lifetime indicating that staining the mesoporous layer in the presence of the bulky co-adsorbers leads to a less dense but more ordered packing of the dye molecules. As a result, the non-radiative intermolecular recombinations are suppressed leading to substantial improvements in the photocurrents and power conversion efficiencies. Our findings are further supported by femtosecond TAS data confirming that the co-adsorbents not only reduced dye aggregation but also accelerated electron injection rates, with a decrease in injection time from 6.4 ps to 4.6 ps. Thus, by highlighting the potential of bile acid derivatives with sterically demanding substituents as effective co-adsorbents, our findings pave the way for facile access to high-performing blue-dye-based semi-transparent hybrid solar cells. This offers promising prospects for dye solar cells in building-integrated photovoltaics.

## Conclusions

In conclusion, we report a series of novel co-adsorbers with sterically demanding substituents offering unique supramolecular features. By fine-tuning the spatial orientation of the substituents we have created co-adsorbers that have an expanded hydrophobic pocket as compared to the conventional CDCA co-adsorber used in the field of dye solar cells. We have demonstrated that attaching bulky substituents at the 3-*beta* position is an effective strategy to mitigate the annihilation effects due to dye aggregation in hybrid solar cells. The effect of increased power conversion is pronounced when using planar sensitizers such as dyenamo blue resulting in the highest current density. The champion dyenamo blue devices based on our co-adsorbers achieved a remarkable PCE of 7.56% under AM1.5G simulated solar illumination, with a significantly improved *J*_SC_ of 13.1 mA cm_2_, a remarkable improvement from existing literature reports. We have thoroughly investigated the effect of the co-adsorbers on the charge transfer characteristics and lateral intermolecular interactions using a combination of impedance and ultrafast spectroscopy techniques. We have discovered that bulky co-adsorbers create an ordered molecular packing of the dye whilst extending the photoluminescence lifetime and enhancing the electron injection rates leading to improved photovoltaic performance. The enhanced bulkiness of the co-adsorber leads to a less-dense monolayer formation of the dye on the mesoporous layer which decreases the area that is active for recombination of conduction band electrons back to the electrolyte. Our findings offer a promising direction towards further improving performances of solar cells that can absorb the invisible near-UV and near-IR parts of the solar spectrum—a feature that can revolutionize building integrated photovoltaics.

## Methods

### Synthesis

#### General procedures

The bile acids, Diisopropil azodicarboxylate (DIAD), Diphenylphosphoryl azide (DPPA), Thionyl Chloride (SOCl2), biphenyl-4-carboxylic acid (IV), and anhydrous THF were bought from Aldrich and used without further purification. Methanol, chloroform, and triethylamine were dried according to literature. Some of the reactions were conducted in oven-dried glassware under a positive pressure of dry nitrogen. The column chromatography was performed on silica gel (Aldrich, technical grade, 70–230 mesh). Thin-layer chromatography was conducted on silica gel plates also from Aldrich using the proper mixture of eluents The synthetic route is shown in Scheme S1

### Synthesis of the compounds

#### Synthesis of the steroid-24-oic acid methyl esters (II)

In a 25 mL one-necked, round-bottomed flask with a magnetic stirring bar, reflux condenser, and calcium chloride trap, 67 mmol of the steroid was added 125 mL of CH3OH and 1 mL of hydrochloric acid (37%), the reaction mix was refluxed by one hour and then cooled first to room temperature and 0 ^∘^C overnight. The mixture was filtered and washed with cold methanol^24^.

#### Synthesis of 3-*β*-aminosteroids (III)

In a 1 L three-necked, round-bottomed flask with a magnetic stirring bar, pressure-equalizing funnel, and dry nitrogen atmosphere, TPP (50 mmol, 13.11 g) and DIAD (50 mmol, 10.11 g) were added to a solution of 50 mmol to the ester of the bile acids in 500 mL of THF. The reaction was stirred for 24 h. Then TPP (50 mmol, 13.11 g) and DIAD (50 mmol, 10.11 g) were added to the mixture and stirs for 4 h, later TPP (50 mmol, 13.11 g) and water (30 mL), the mixture was stirred for 4 days. The THF was evaporated in vacuo, and the residue was solved with ethyl acetate and dried using sodium sulfate and then the solvent was evaporated in vacuo. The crude was purified by chromatography on silica gel. A mixture of methanol and triethyl amine (95:5) was used as eluent^25^.

#### Synthesis of the acyl chloride (IV)

In an oven-dried 50-mL three-necked, round-bottomed flask with a magnetic stirring bar, reflux condenser, and calcium chloride trap, 0,5 g (0.25 mmol) of the biphenyl-4-carboxylic acid was added 20 mL of SOCl2, the reaction mixture was refluxed for 3 h, then the SOCl2 was evaporated in vacuo and the residue was dissolved in 5 mL of chloroform for the next step^26^.

#### Synthesis of the 3*β*-bile acid-biphenyl derivatives (V)

In an oven-dried 50-mL three-necked, round-bottomed flask with magnetic stirring bar, a solution of 1 g of the amino steroid in 25 mL of CHCl3 as solvent was added 2 mL of TEA, under nitrogen atmosphere at room temperature and later cooled to 0 ^∘^C. Then the acyl chloride was added dropwise to the mixture. The reaction mixture was stirred for 24 h at room temperature. The crude mixture was concentrated and purified by silica gel chromatography with ethyl acetate and hexane as eluents in a ratio of 7:3 for the BIAC01C, 1:1 for the BIAC02D BIAC04U and BIAC05Q, and 4:6 for the BIAC03L^26^.

#### Synthesis of the final mixed dimers (VI)

In a 25 mL one-necked, round-bottomed flask with magnetic stirring bar and reflux condenser, a solution of 100 mg of the amino steroid in 5 mL of KOH 1 molL^−^1 was refluxed for 1 h. The reaction was checked using TLC. The reaction crude was evaporated and then dissolved in 100 mL of distilled water. Hydrochloric acid (37%) was added dropwise to the solution until the pH 2. The solid was filtrated and washed with distilled water until pH 5. The solid was dried in vacuum oven overnight, and, if necessary, purified by column chromatography with ethyl acetate and hexane as eluents in a ratio of 7:3 for the BIAC01C, 1:1 for the BIAC02D, BIAC04U, and BIAC05Q and 4:6 for the BIAC03L^26^.

### Dye-sensitized solar cell fabrication

The fabrication of DSCs was done as previously reported^27^. All FTO glasses were cleaned with Hellmanex. water and ethanol using ultrasonication in a water bath for 15 min each followed by rinsing and drying in air. The working electrodes of FTO were coated with a blocking layer (~50 nm) using spray pyrolysis at 450 ^∘^C. The precursor solution used for spray pyrolysis was composed of anhydrous 2-propanol (9 mL), titanium diisopropoxide bis(acetylacetonate) (600 μL) and acetylacetone (400 μL). The resultant compact layer was sintered at 450 ^∘^C for 60 min. A mesoporous TiO_2_ layer was screen printed (4 μm) using the Dyesol/GreatCellSolar (Queanbeyan, Australia) DSL 30-NR titania paste. After drying at 120 ^∘^C for 5 min, a scattering layer (Dyesol/GreatCellSolar WER2-0) was screen-printed on top of the mesoporous film (4 μm) followed by a 30 min sintering steo at 500 ^∘^C. After cooling the substrates were immersed in a 26 mM TiCl_4_ aqueous solution for 30 min at 70 ^∘^C and then sintered again at 500 ^∘^C for 30 min. Dye solution was prepared with 0.075 mM Dyenamo Blue (Dyenamo, Stockholm, Sweden) and 0.2 mM of co-adsorbants CDCA (Chenodeoxycholic acid), BIAC01C, 03L, 05Q in a 1:1 acetonitrile:tert-butanol mixture. Dye solutions were prepared with the co-adsorbers in the concentration ratio (1:0.5, 1:2, 1:10). For the solutions with the novel co-adsorbers tested in this work, the dye bath was subjected to ultrasonication to ensure complete dissolution of the additives. The above-prepared TiO_2_ substrates were immersed in the dye solutions for 16 h before being assembled with the counter electrodes. The counter electrodes were manufactured using an electropolymerization of (3,4-ethylenedioxythiophene) from a 0.01 mM aqueous solution with 0.1 M sodium dodecyl sulfate as previously reported. For the Dyenamo blue-based cells, the electrolyte comprised 0.2 M Co(bpy)_3_(TFSI)_2_ and 0.05 M Co(bpy)_3_(TFSI)_3_. All electrolytes were prepared in acetonitrile with additions of 0.1 M LiTFSI and 0.6 M 4-tert-butylpyridine.

### Characterization of the solar cells

#### Current-voltage measurements

Current-voltage measurements under AM 1.5G illumination were carried out in ambient air using a Sinus-70 solar simulator (Wavelabs Solar Metrology Systems GmbH). The irradiance (100 mW cm^−2^) was calibrated with a certified silicon diode (Fraunhofer). An X200 source meter (Ossila, Sheffield, UK) was used to assess the solar cell performance (scan speed 25 mVs^−1^, 5 mV step size). A circular mask was employed to confine the active solar cell area to 0.196 cm^2^. Shown device performance metrics represent averages of four cells at all times, pending reasonable error margins of the manual cell fabrication.

#### Incident photon-to-current conversion

IPCE spectra were recorded with a Xenon light source (10 mW cm^−2^) and a CM110 monochromator (Bentham). The setup was calibrated with a certified silicon reference cell (Fraunhofer ISE, Munich, Germany). Photocurrents were integrated based on the spectral distribution of sunlight AM 1.5G.

#### Transient photovoltage and charge collection measurements

Electron recombination lifetimes were investigated using the dye-sensitized solar cell Toolbox (Dyenamo, Sweden). The solar cell was illuminated with a 1 W white LED. Kinetics in the solar cell were probed by applying slow 10 Hz square-wave modulations on top of a base light intensity. The solar cell voltage response was tracked in real time the measurement was repeated until a noise threshold was met; at which point the traces were fitted with first-order kinetic models and a single decay time constant from the compiled traces was extracted. The measurements were run across a range of light intensities. The accumulated charge in the photoanodes of the DSC devices was measured by illuminating the cells at different light intensities at an open circuit; then, the light was turned off, the potential simultaneously switched to short circuit, and the current integrated over time. The collected charge was converted to charge density assuming a 6 mm diameter of the circular aperture, 4 μm anode thickness, and 0.63 porosity.

#### Time-resolved Photoluminescence spectroscopy

Photoluminescence lifetimes were recorded at the emission centre of 760 nm on an Edinburgh FLS980 spectrometer. Samples were excited with an EPL-475 (472 nm, 61 ps pulse width) picosecond laser, and measured with a time-correlated single photon counting module and a Hamamatsu R928P photomultiplier tube. Lifetime fits of the photoluminescence intensity I were extracted directly in the F980 software with numerical data reconvolution based on Marquardt–Levenberg algorithm, with exponential traces as

$$I=\mathop{\sum}\limits_{i}{B}_{{{\rm{i}}}}{e}^{{\tau }^{-1}{{\rm{t}}}}$$

with amplitude factors B_i_ and time constants *τ*_i_. To present the traces, the counts of photons before time zero were averaged to serve as a baseline. then, the relative amplitudes in all scans were rescaled between the common baseline and the peak intensity normalized to 1.

#### Electrochemical Impedance spectroscopy

Electrochemical impedance spectra were recorded using a PGSTAT12 potentiostat (Autolab) in the frequency range from 100 kHz to 0.1 Hz at different DC bias potentials around the maximum power point, modulating the voltage by 10 mV. The DSC devices were illuminated with a white LED for impedance analysis and fitted to Bisquert’s transmission-recombination line with an adapted version of the impedance fitting tool available on Z-view software.

#### Dye loading on the TiO_2_ surface

The percentage of dye molecules adsorb on TiO_2_ surface relative to the reference device (blank) was obtained following the dye-desorption method. After dipping the TiO_2_ substrates in the different co-adsorbate/dye solutions, the molecules loaded on the semiconductor were then desorbed by dipping them in a solution of tetrabutylammonium hydrozide 0.1 mol L^−1^, followed by UV-Vis spectroscopic determination of dye concentration by Beer-Lambert law with the extinction coefficient of the peak absorption the dye.

#### Transient absorption spectroscopy

TAS measurements were performed using an Ultrafast Systems TAS Spectrometer. Samples were pumped with Ti:Sapphire laser at 1 kHz (800 nm source pump). Excitation wavelength was selected using an optical parametric amplifier. For ultrafast measurements, a portion of the pump beam was routed through a sapphire crystal to achieve white light continuum to act a probe beam. Excitation of 490 nm was used with a pump power of 100 μW. The pump power was varied to check for linearity and minimized to limit dye photobleaching. The pulse width is 100 fs. Four spectra were taken per sample and averaged to achieve the presented spectra.

### Reporting summary

Further information on research design is available in the Nature Portfolio Reporting Summary linked to this article.

## Supplementary information

Supporting Information

Reporting Summary
