# Supplementary material for: Bile acid derivatives as novel co-adsorbents for enhanced performance of blue dye-sensitized solar cells
Source: Commun Chem. 2025 Mar 10;8:75. doi: 10.1038/s42004-025-01433-1 (PMC11894181; doi:10.1038/s42004-025-01433-1)
Supplement: Supplementary file 1 — Supporting Information [file 42004_2025_1433_MOESM1_ESM.pdf]

Supporting Information

Bile Acid Derivatives as Novel Co-adsorbents for Enhanced Performance of Blue Dye-Sensitized Solar Cells

Kezia Sasitharan,[1] Allan J. Mora Abarca,[2] Fabio Cucinotta,[1] Leslie W. Pineda,[2] Victor Hugo Soto Tellini,[2] and Marina Freitag,[1,*]

[1] School of Natural and Environmental Science, Bedson Building, Newcastle University, NE1 7RU Newcastle upon Tyne, UK
[2] Centro de Investigación en Electroquímica y Energiá Química (CELEQ), Universidad de Costa Rica, 11501-2060, Costa Rica

Contents

1 Synthesis of coadsorbers 1
2 Structural Information 1
3 Optical properties 35
4 Photovoltaic performance 36
5 Photoluminescence spectrocopy 40
6 Transient absorption spectroscopy 40

1 Synthesis of coadsorbers

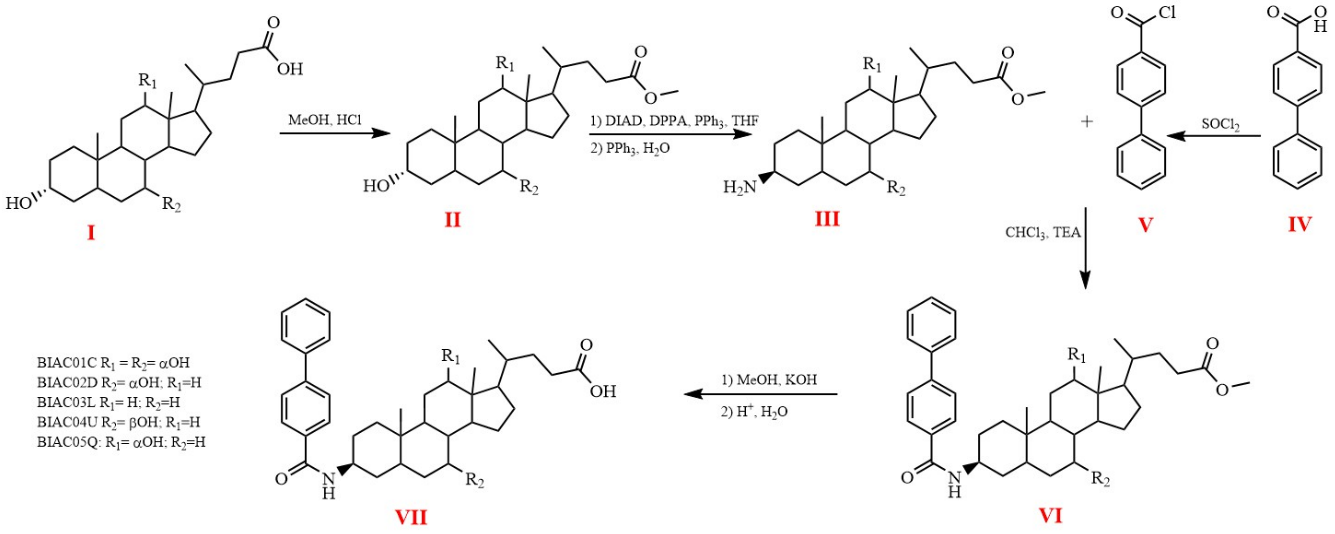

Scheme S1. Schematic of synthesis of the new compounds

2 Structural Information

NMR spectra

NMR Samples were dissolved in DMSO-D6. Data were collected using an Ascend 600MHz NMR equpment controlled by a Bruker Advance III console and analyzed with MestreNova version 14.2.

H:	δ-H/J:BIAC01C	δ-H/J: BIAC02D	δ-H/J: BIAC05Q	δ-H/J: BIAC04U	δ-H/J: BIAC03L
1	1.43; 1.33; m	1.14; 1.34; m	1.49; 1.36; m	1.47; 1.40; m	1.48; 1.37; m
2	1.63; 1.57; m	1.62; 1.56; m	1.57; 1.54; m	1.56; m	1.58; m
3	4.08; m	4.19; m	4.08; m	4.12; m	4.18; m
4	2.60; 1.49; m	2.03; 1.38; m	2.56; 1.50; m	1.87; 1.50; m	2.03; 1.37; m
5	1.73; m	1.77; m	1.73; m	1.32; m	1.77; m
6	1.85; 1.34; m	1.83; 1.13; m	1.87; 1.37; m	1.70; 1.38; m	1.85; 1.13; m
7	3.65, m	1.36; 1.08; m	3.66; m	3.33; m	1.37; 1.07; m
8	1.38; m	1.37; m	1.38; m	1.41; m	1.37; m
9	2.19, m	1.85; m	1.80; m	1.82; m	2.08; m
10	-	-	-	-	-
11	1.43; m	1.40; m	1.41; 1.24; m	1.38; 1.23; m	1.37; 1.20; m
12	3.80; m	3.80; m	1.92; 1.15; m	1.94; 1.16; m	1.93; 1.19; m
13	-	-	-	-	-
14	2.00; m	1.59; m	1.44; m	1.18; m	1.42; m
15	1.66; 0.99; m	1.53; 1.01; m	1.68; 1.01; m	1.86; 1.36; m	1.80; 1.25; m
16	1.73; 1.18; m	1.75; 1.18; m	1.80; 1.25; m	1.75; 1.21; m	1.55; 1.03; m
17	1.81; m	1.79; m	1.10; m	1.03; m	1.09; m
18	0.61; s	0.62; s	0.63; s	0.64; s	0.63; s
19	0.90; s	0.93; s	0.93; s	0.96; s	0.96; s
20	1.30; m	1.31; m	1.39; m	1.36; m	1.37; m
21	0.94; 6.48	0.93; m	0.84; 6.53	0.89; 6.49	0.89; 6.56
22	1.67; 1.22; m	1.66; 1.21; m	1.69; 1.20; m	1.69; 1.21; m	1.68; 1.20; m
23	2.23; 2.11; m	2.23; 2.11; m	2.23; 2.12; m	2.23; 2.11; m	2.23; 2.11; m
24	-	-	-	-	-
OH7	4.08; 3.24	-	4.15; 3.38	3.88; 6.67	-
OH12	4.14; 3.43	4.19; 4.50	-	-	-

Table S1. Chemicals shift of protons (δ: ppm) and J values (Hz) of the synthesized derivatives

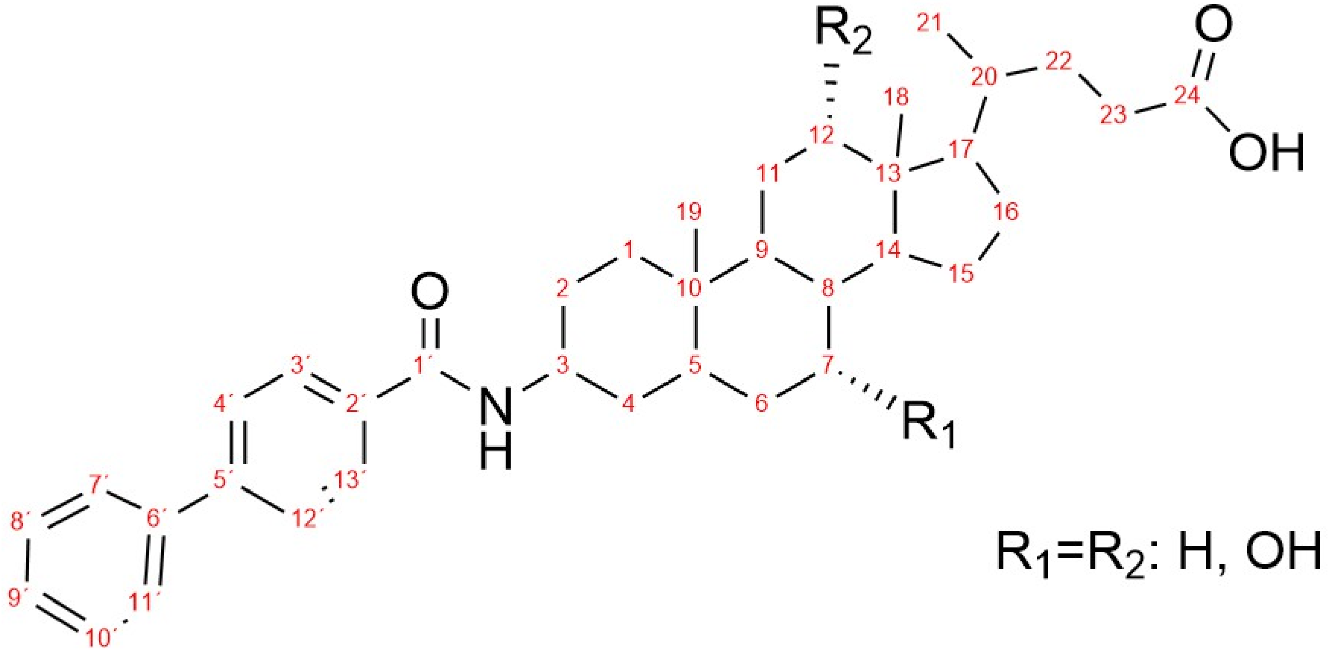

Scheme S2. Structure of the new compounds

C:	δ-C/J:BIAC01C	δ-C/J: BIAC02D	δ-C/J: BIAC05Q	δ-C/J: BIAC04U	δ-C/J: BIAC03L
1	31.1	31.1	31.3	31.0	31.2
2	24.8	24.8	24.8	24.8	24.8
3	46.3	46.2	46.3	45.9	46.1
4	33.6	30.5	33.5	31.6	30.6
5	36.9	37.0	36.8	43.5	37.0
6	34.8	26.9	34.8	37.7	26.9
7	66.9	26.2	66.8	71.1	26.3
8	40.0	36.0	39.5	38.8	35.3
9	26.3	32.9	32.4	37.8	31.4
10	35.2	34.6	35.4	34.6	35.1
11	29.2	29.2	21.0	21.6	21.3
12	71.6	71.7	39.9	40.3	40.2
13	46.3	46.5	42.4	43.6	42.7
14	41.9	48.0	50.5	56.3	39.8
15	23.2	24.0	23.6	27.2	27.9
16	27.7	27.6	28.2	28.6	24.3
17	46.7	46.7	56.0	55.3	56.0
18	12.9	12.9	12.2	12.6	12.4
19	23.2	23.8	23.5	24.0	24.0
20	35.5	35.5	35.6	35.4	35.7
21	17.5	17.4	18.7	18.8	18.7
22	31.1	31.2	31.3	31.2	31.2
23	31.1	31.3	31.3	31.3	31.2
24	175.4	175.4	175.4	175.4	175.8

Table S2. Chemicals shift of carbons in the sinthetized derivatives (δ: ppm)

H:	δ-H/J:BIAC01C	δ-H/J: BIAC02D	δ-H/J: BIAC05Q	δ-H/J: BIAC04U	δ-H/J: BIAC03L
1´	-	-	-	-	-
2´	-	-	-	-	-
3´	7.91; 8.33	7.91; 8.43	7.90; 8.44	7.91; 8.23	7.91; 8.38
4´	7.74; 8.41	7.75; 8.48	7.74; 8.51	7.75; 8.23	7.74; 8.63
5´	-	-	-	-	-
6´	-	-	-	-	-
7´	7.72; 8.46	7.72; 8.50	7.72; 8.50	7.72; 7.61	7.72; 8.49
8´	7.50; 7.60	7.50; 7.58	7.50; 7.55	7.50; 7.61	7.50; 7.58
9´	7.41; 7.30	7.42; 7.40	7.41; 7.37	7.41; 7.51	7.41; 7.47
10´	7.50; 7.60	7.50; 7.58	7.50; 7.55	7.50; 7.61	7.50; 7.58
11´	7.72; 8.46	7.72; 8.50	7.72; 8.50	7.72; 7.61	7.72;
12´	7.74; 8.41	7.75; 8.48	7.74; 8.51	7.75; 8.23	7.74; 8.63
13´	7.91; 8.33	7.91; 8.43	7.90; 8.44	7.91; 8.23	7.91; 8.38
N-H	7.95; 6.35	8.03; 6.53	7.96; 6.29	8.03; 6.50	8.06; 6.56

Table S3. Chemicals shift of protons (δ:ppm) and principal J values of aa´bb´ aromatic protons (Hz) in the biphenyl moiety of the synthesized derivatives

C:	δ-C: Cholic	δ-C: Deoxycholic	δ-C: Chenodeoxycholic	δ-C: Ursodeoxycholic	δ-C: Litocholic
1´	166.7	166.8	166.6	166.6	166.9
2´	134.6	134.6	134.8	134.6	134.5
3´	128.6	128.8	128.7	128.8	128.8
4´	126.7	126.7	126.7	126.7	126.7
5´	142.9	142.9	143.0	145.0	142.9
6´	139.8	139.9	140.0	139.8	139.8
7´	127.5	127.3	127.3	127.3	126.3
8´	129.5	129.4	129.5	128.5	129.5
9´	128.7	128.4	128.7	128.4	128.5
10´	129.5	129.4	129.5	129.5	129.5
11´	127.5	127.3	127.3	127.3	126.3
12´	126.7	126.7	126.7	126.7	126.7
13´	128.6	128.8	128.7	128.8	128.8

Table S4. Chemicals shift of carbons in the biphenyl moiety of the sinthetized derivatives (δ: ppm)

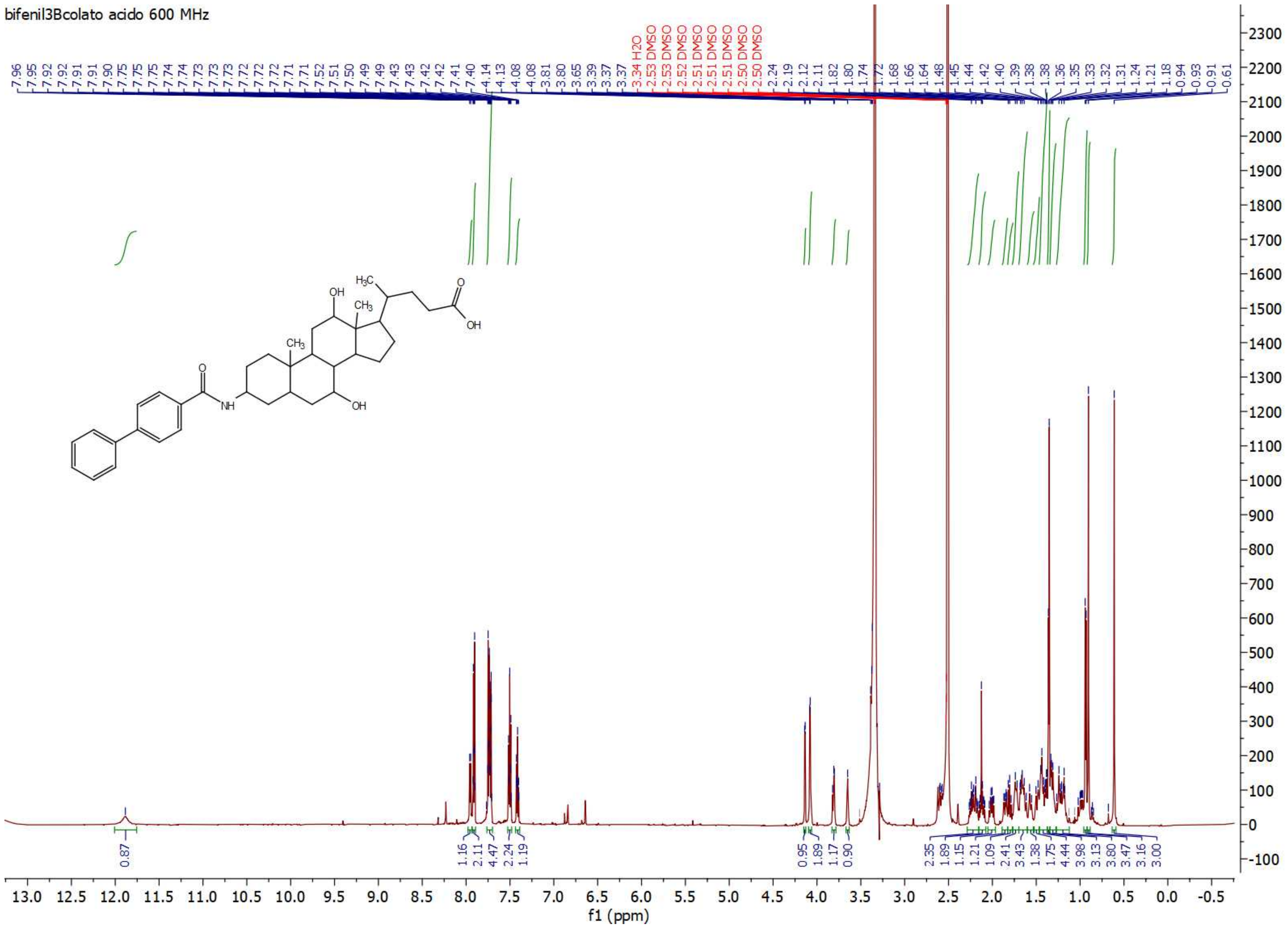

Figure S1. 1H-NMR spectrum of BIAC01C in DMSO-D6 (600 MHz).

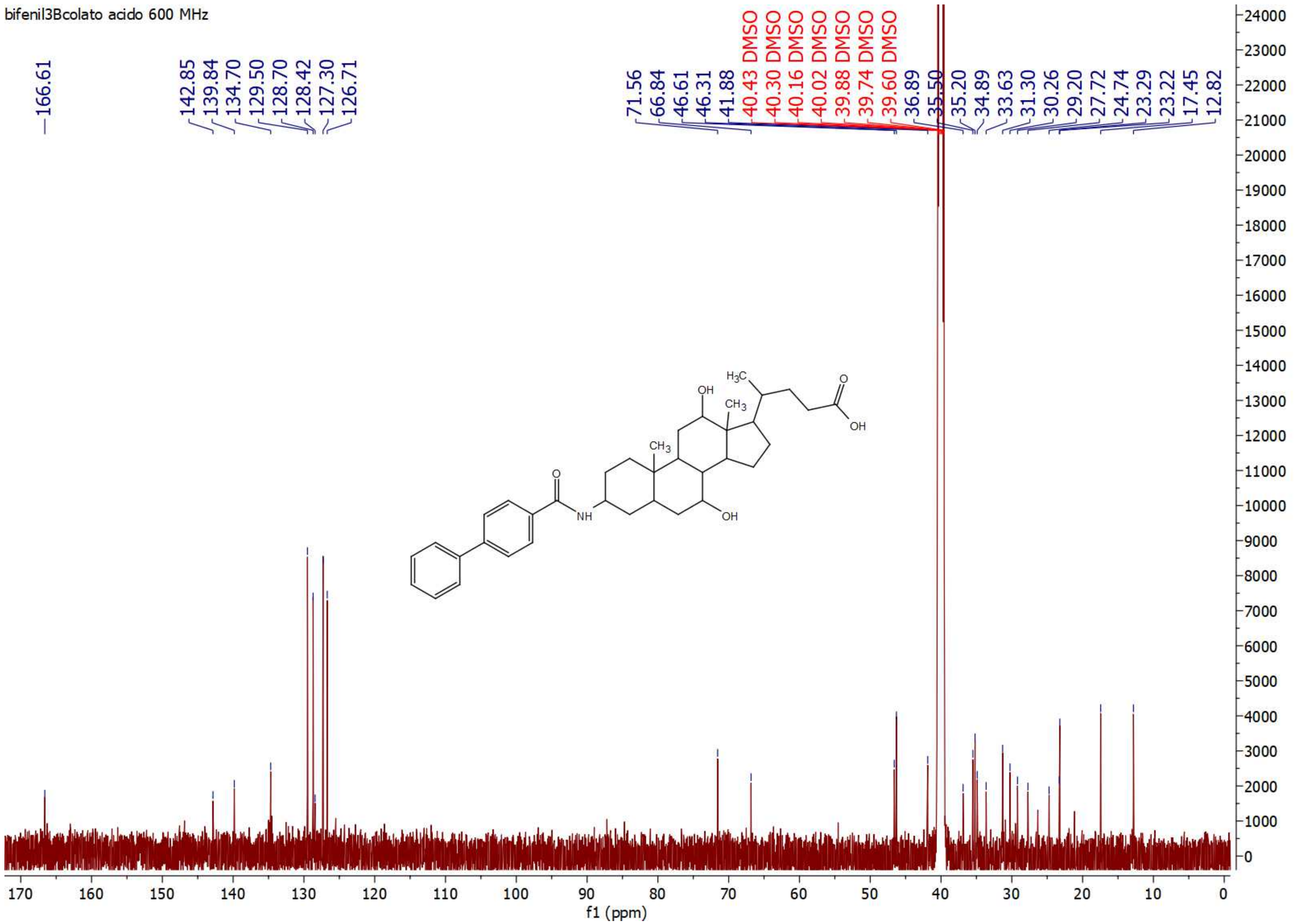

Figure S2. 13C-NMR spectrum of BIAC01C in DMSO-D6 (600 MHz)

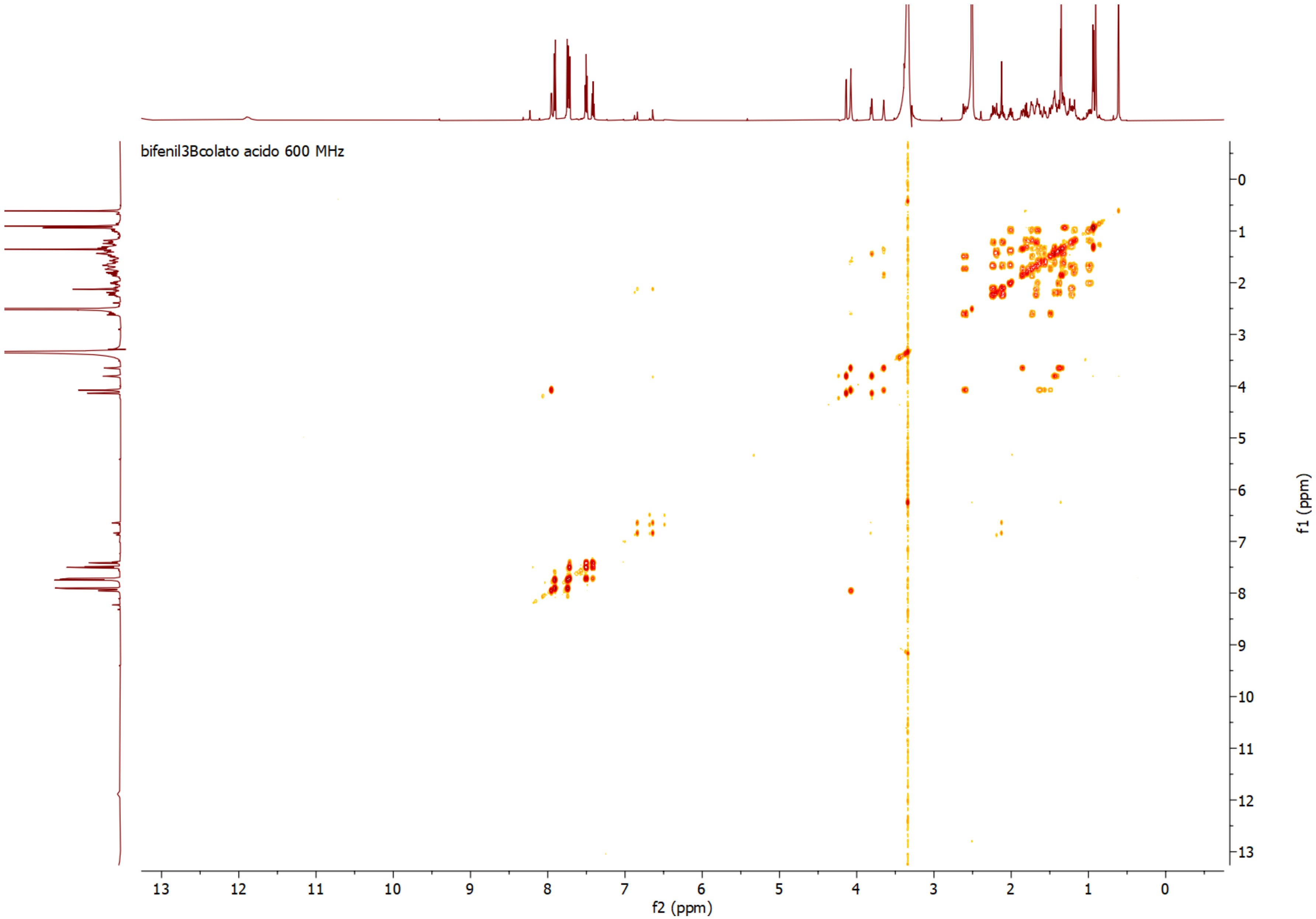

Figure S3. COSY NMR spectrum of BIAC01C in DMSO-D6 (600 MHz)

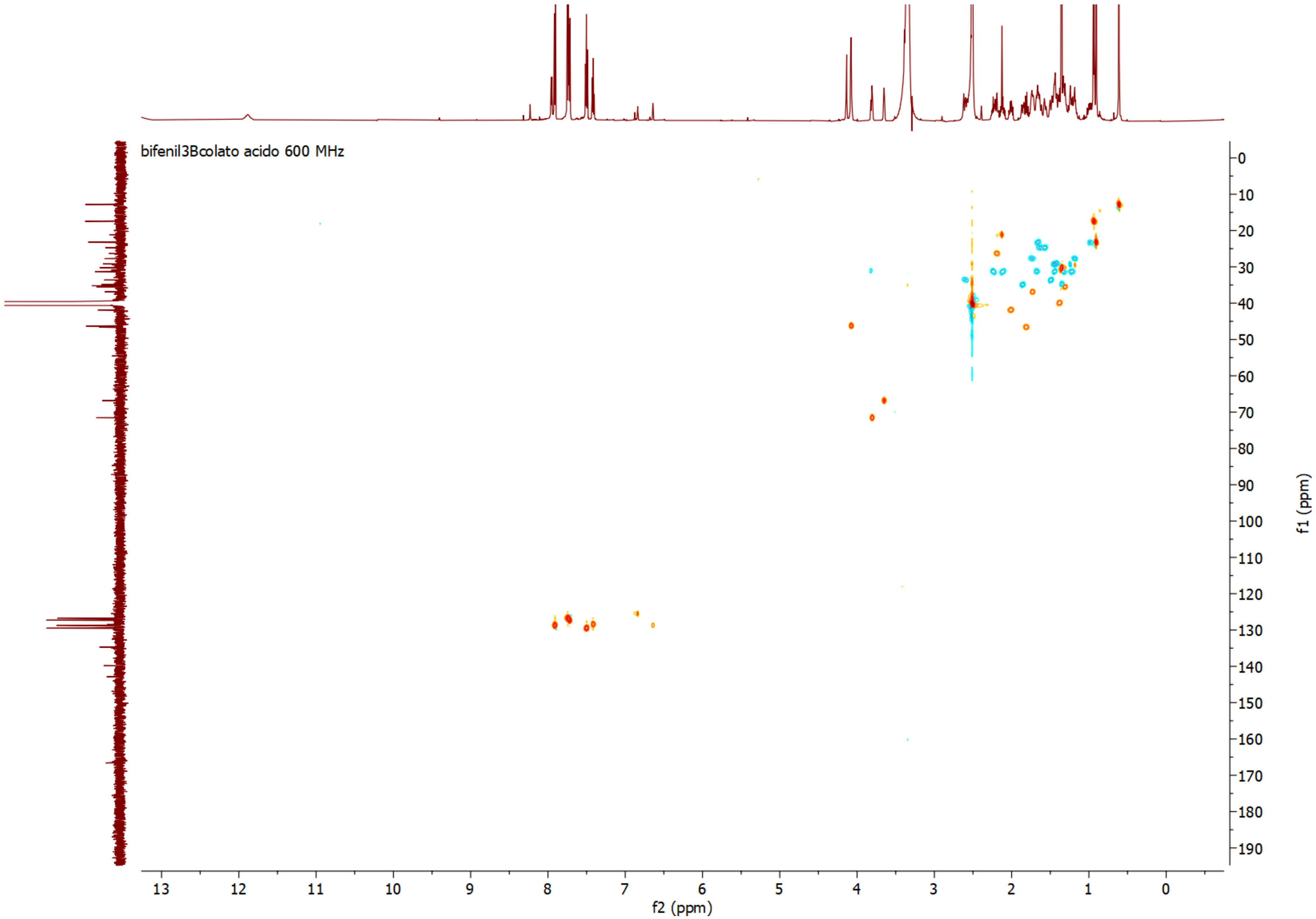

Figure S4. HSQC NMR spectrum of BIAC01C in DMSO-D6 (600 MHz)

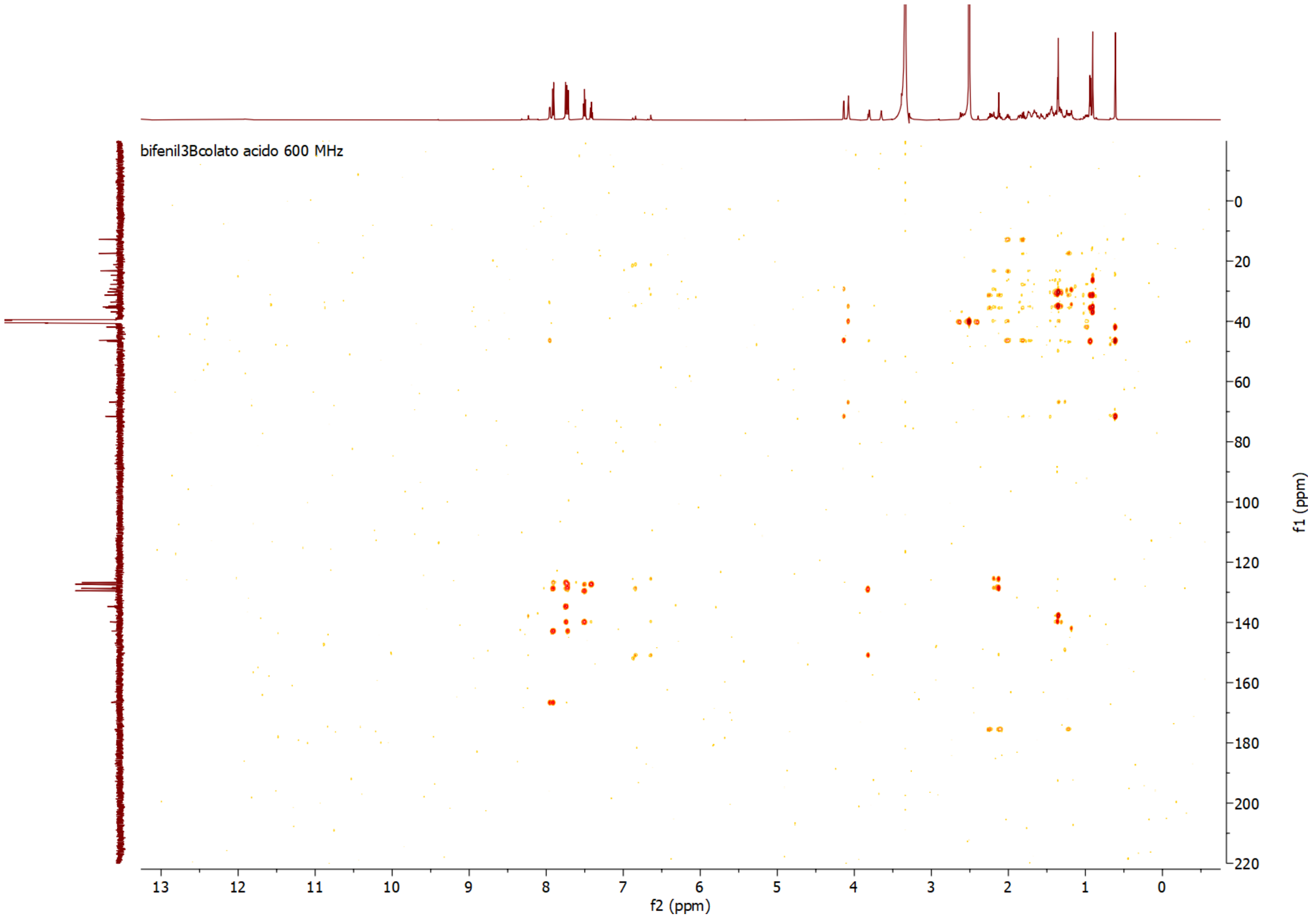

Figure S5. HMBC NMR spectrum of BIAC01C in DMSO-D6 (600 MHz)

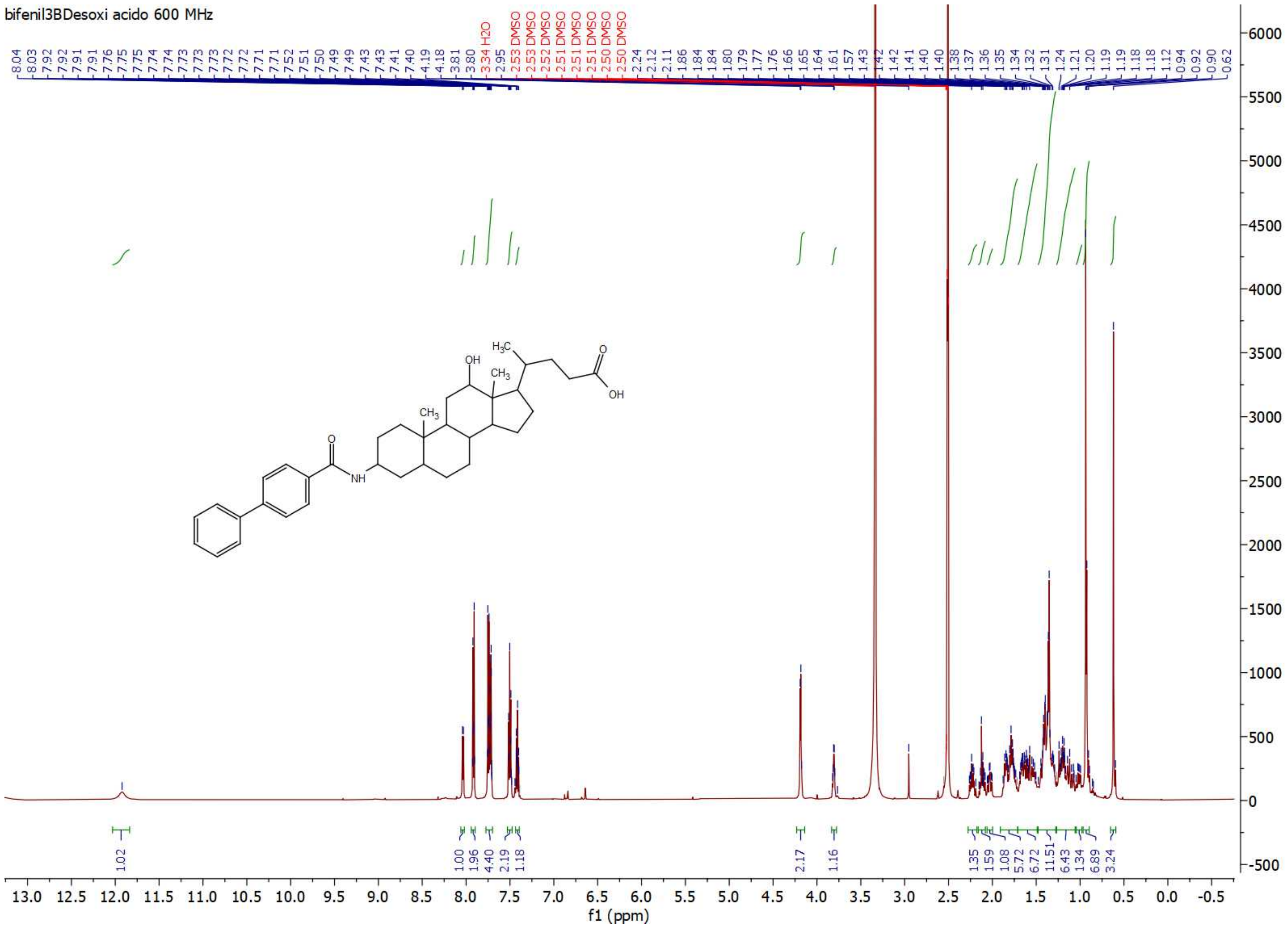

Figure S6. 1H-NMR spectrum of BIAC02D in DMSO-D6 (600 MHz).

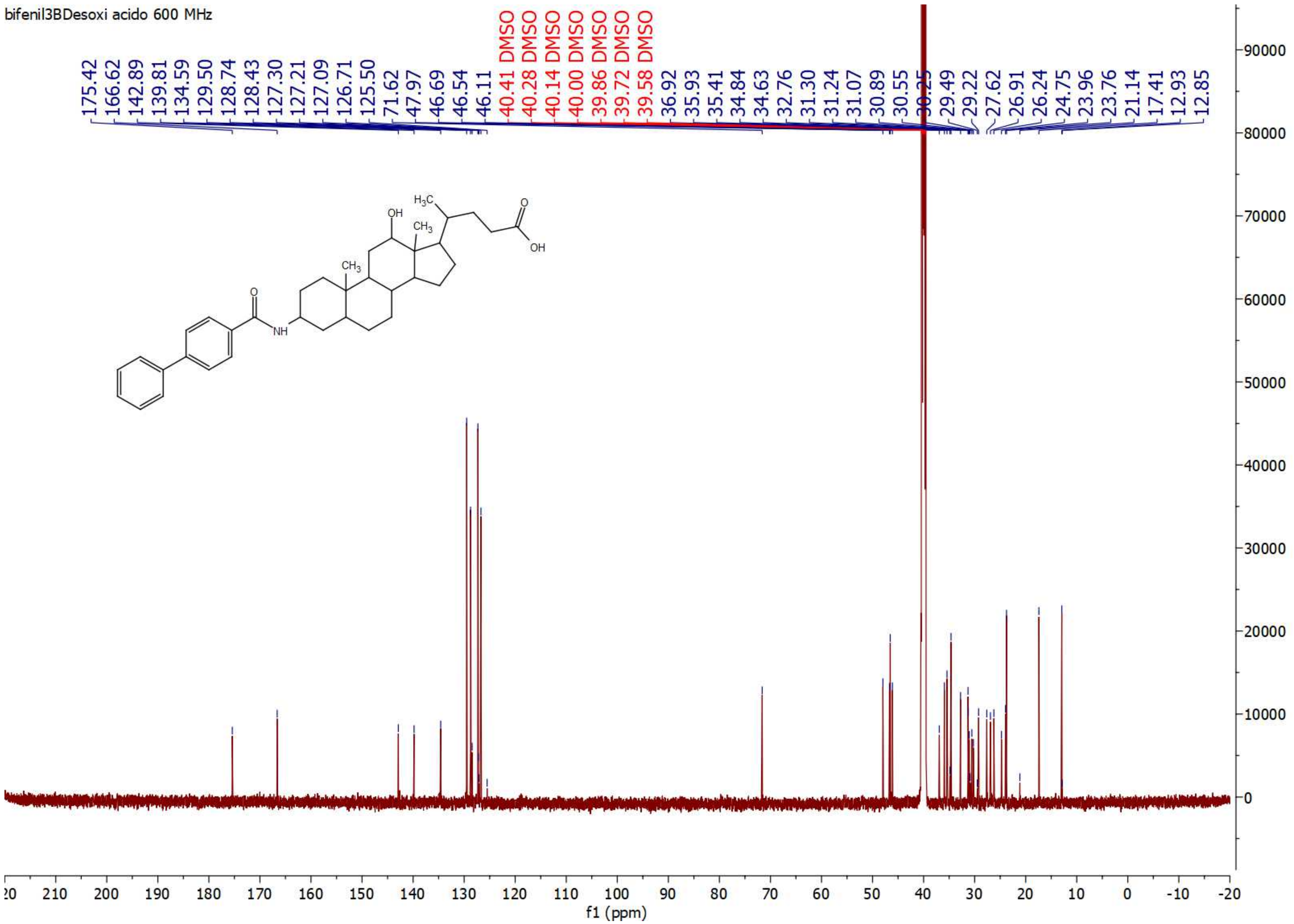

Figure S7. 13C-NMR spectrum of BIAC02D in DMSO-D6 (600 MHz).

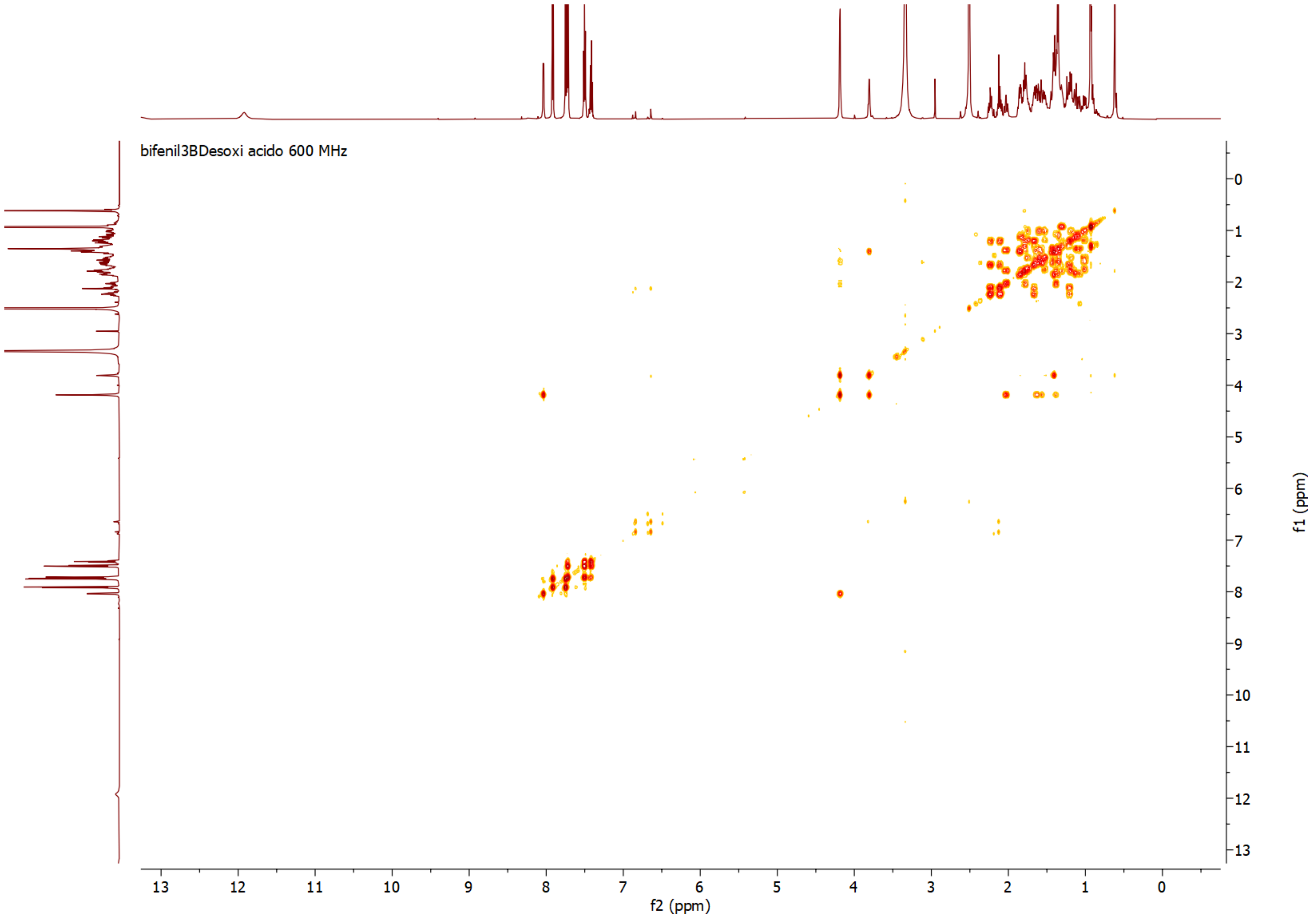

Figure S8. COSY NMR spectrum of BIAC02D in DMSO-D6 (600 MHz).

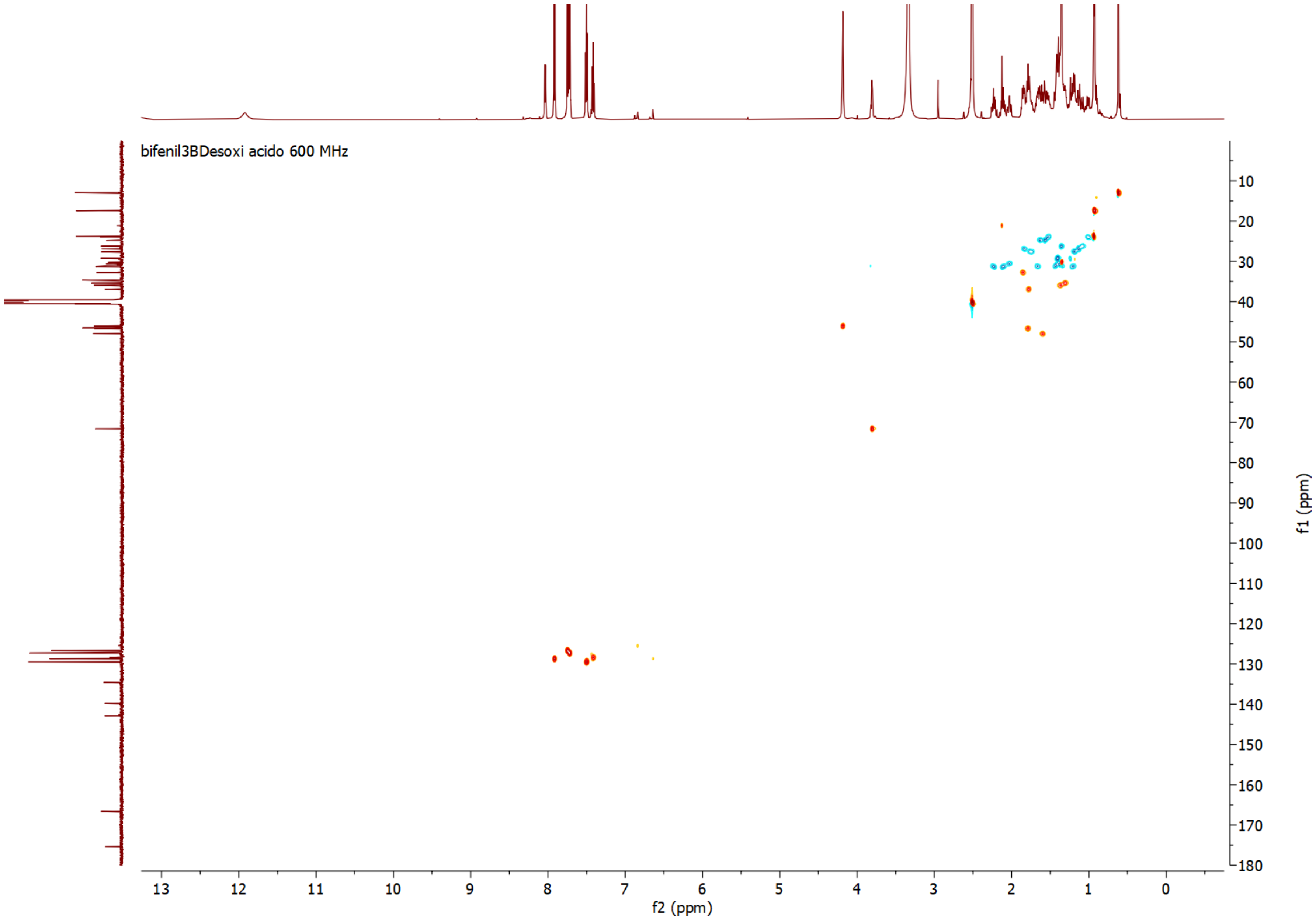

Figure S9. HSQC NMR spectrum of BIAC02D in DMSO-D6 (600 MHz).

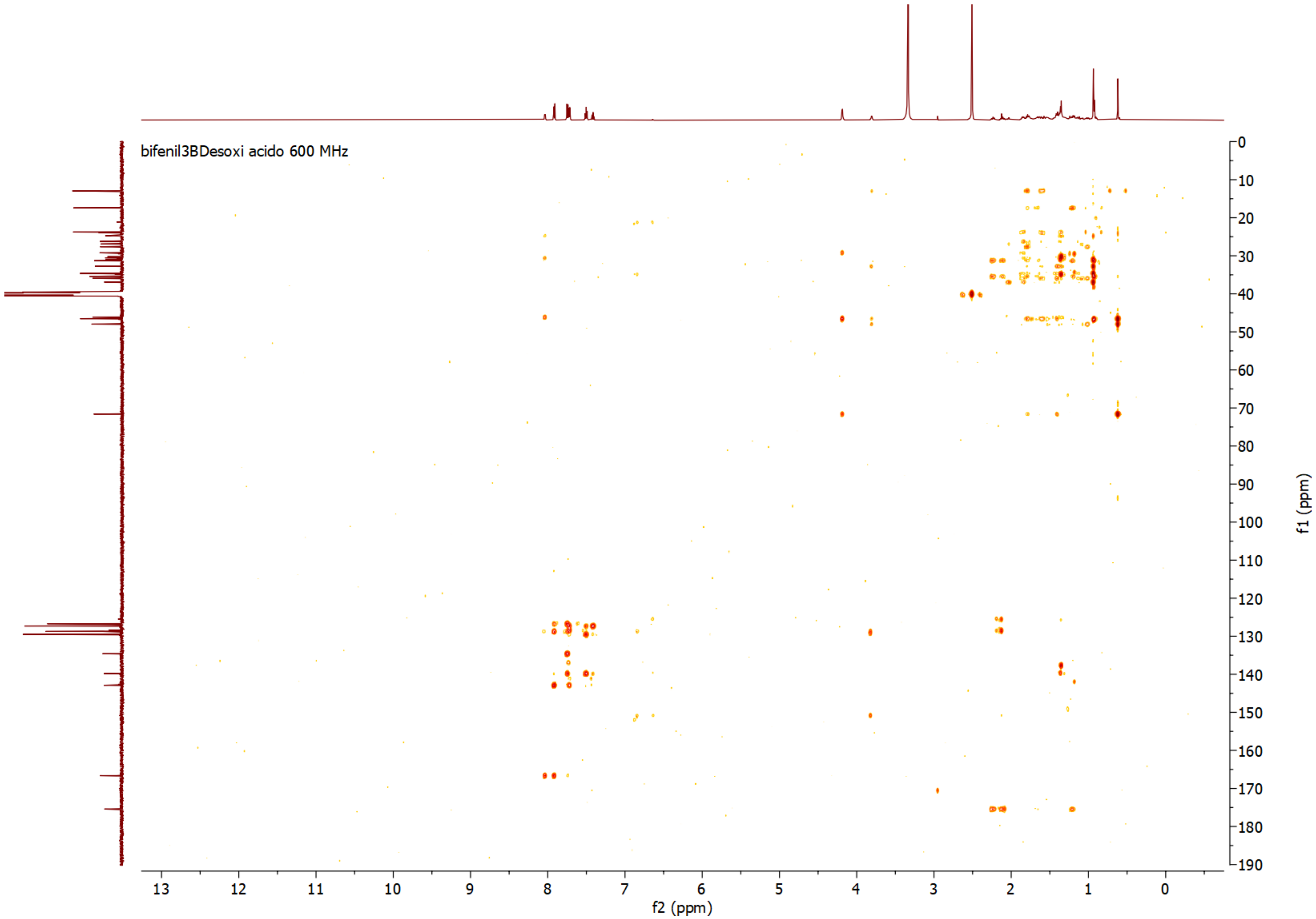

Figure S10. HMBC NMR spectrum of BIAC02D in DMSO-D6 (600 MHz).

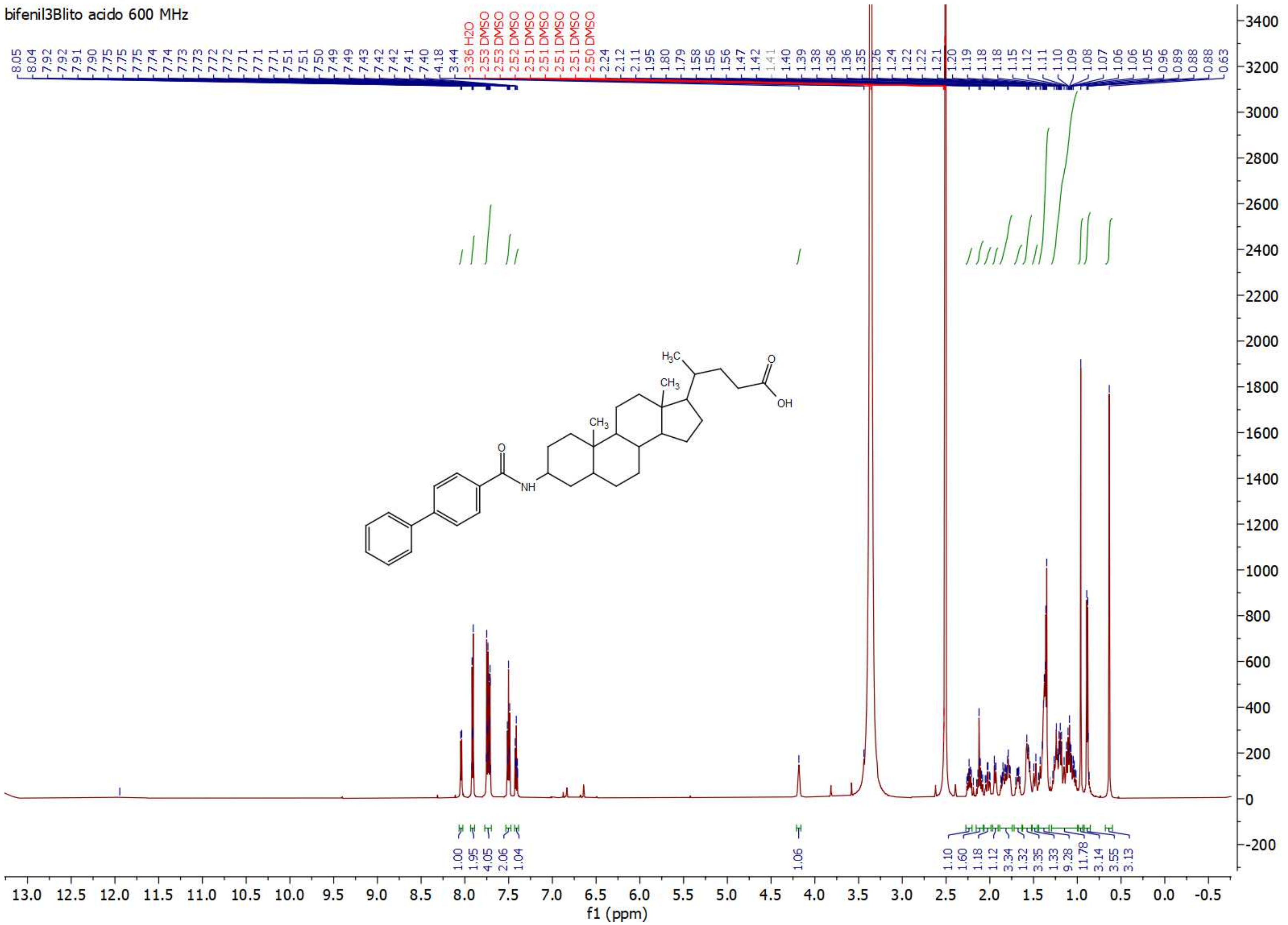

Figure S11. 1H-NMR spectrum of BIAC03L in DMSO-D6 (600 MHz).

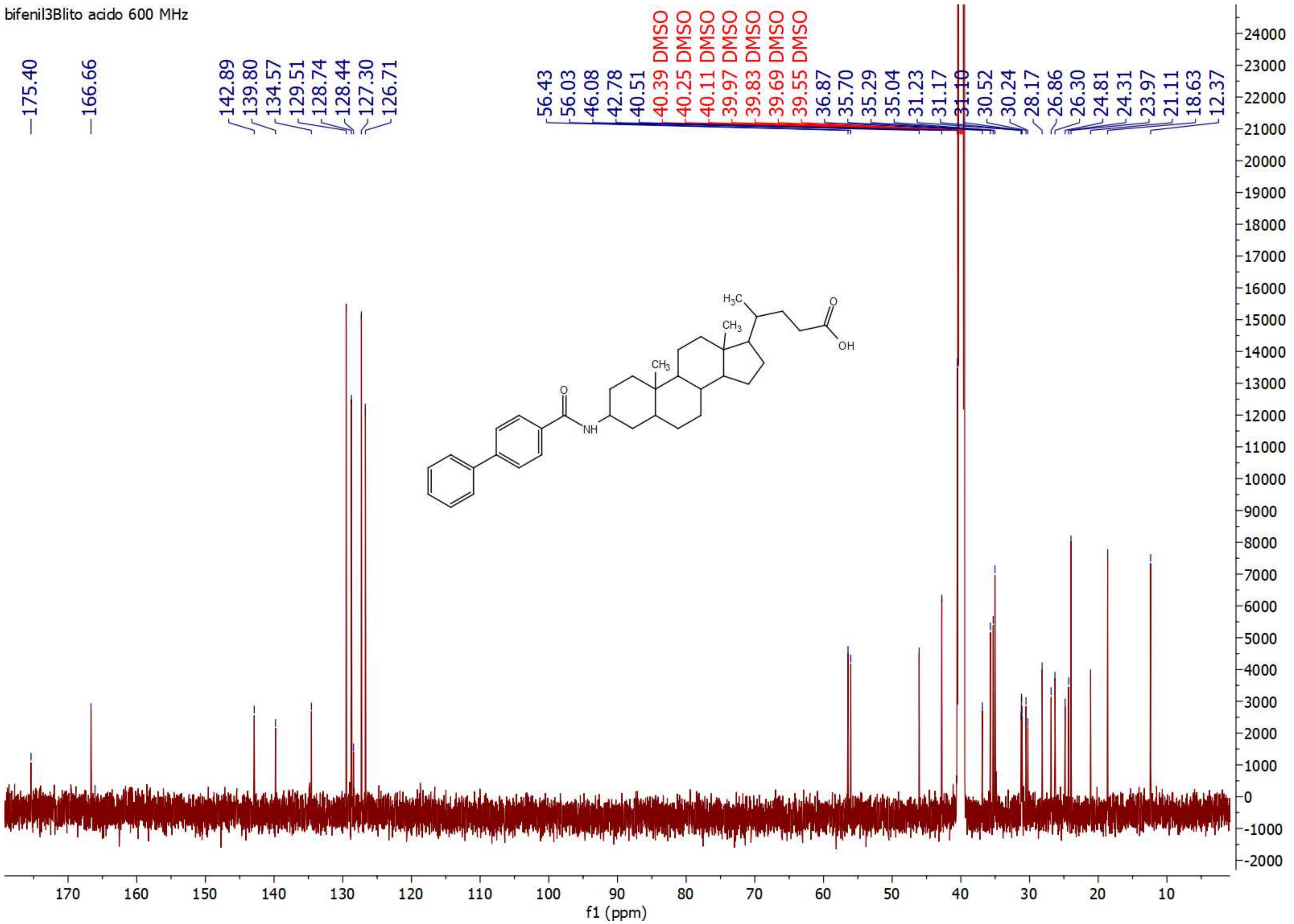

Figure S12. 13C-NMR spectrum of BIAC03L in DMSO-D6 (600 MHz).

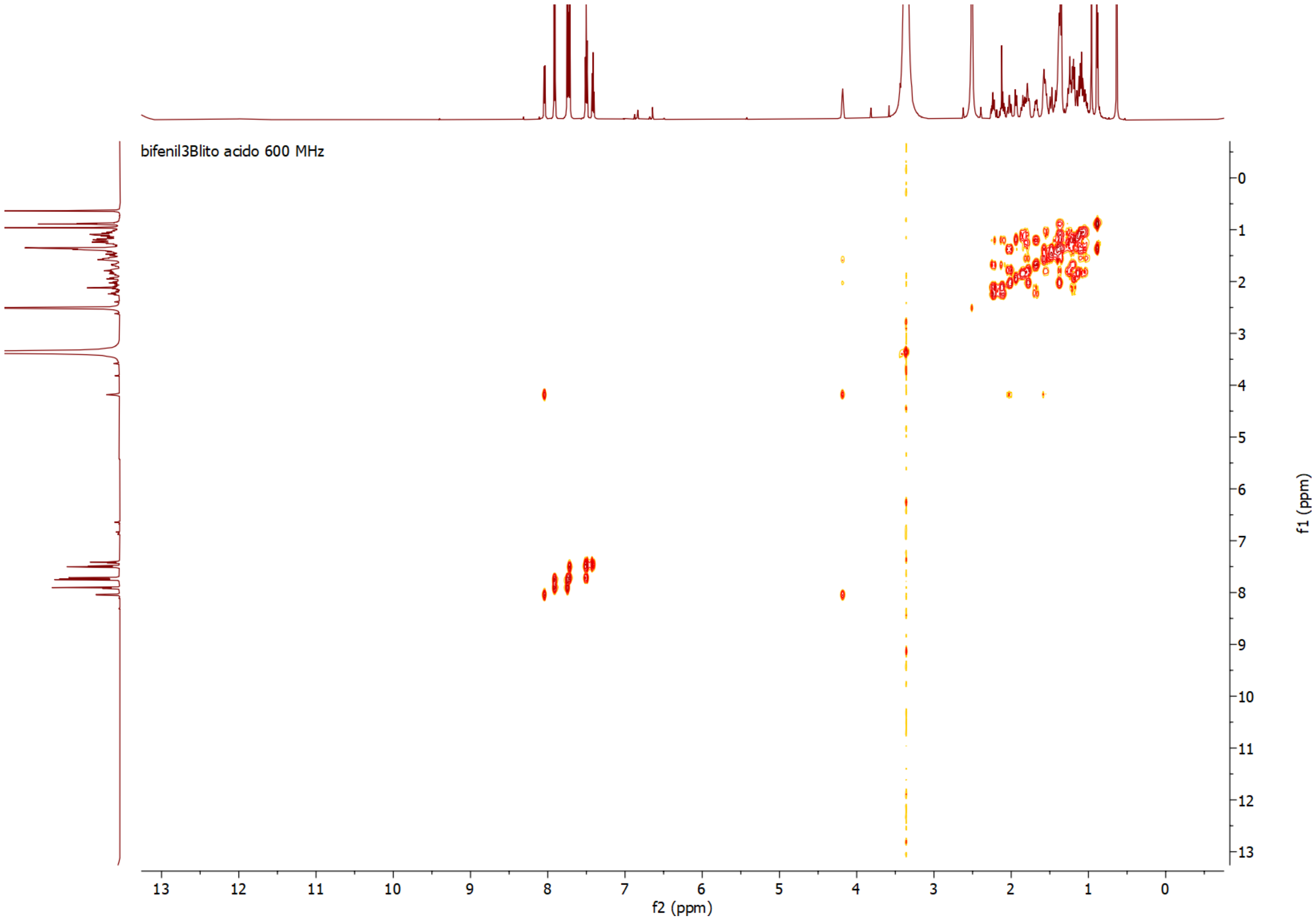

Figure S13. COSY NMR spectrum of BIAC03L in DMSO-D6 (600 MHz).

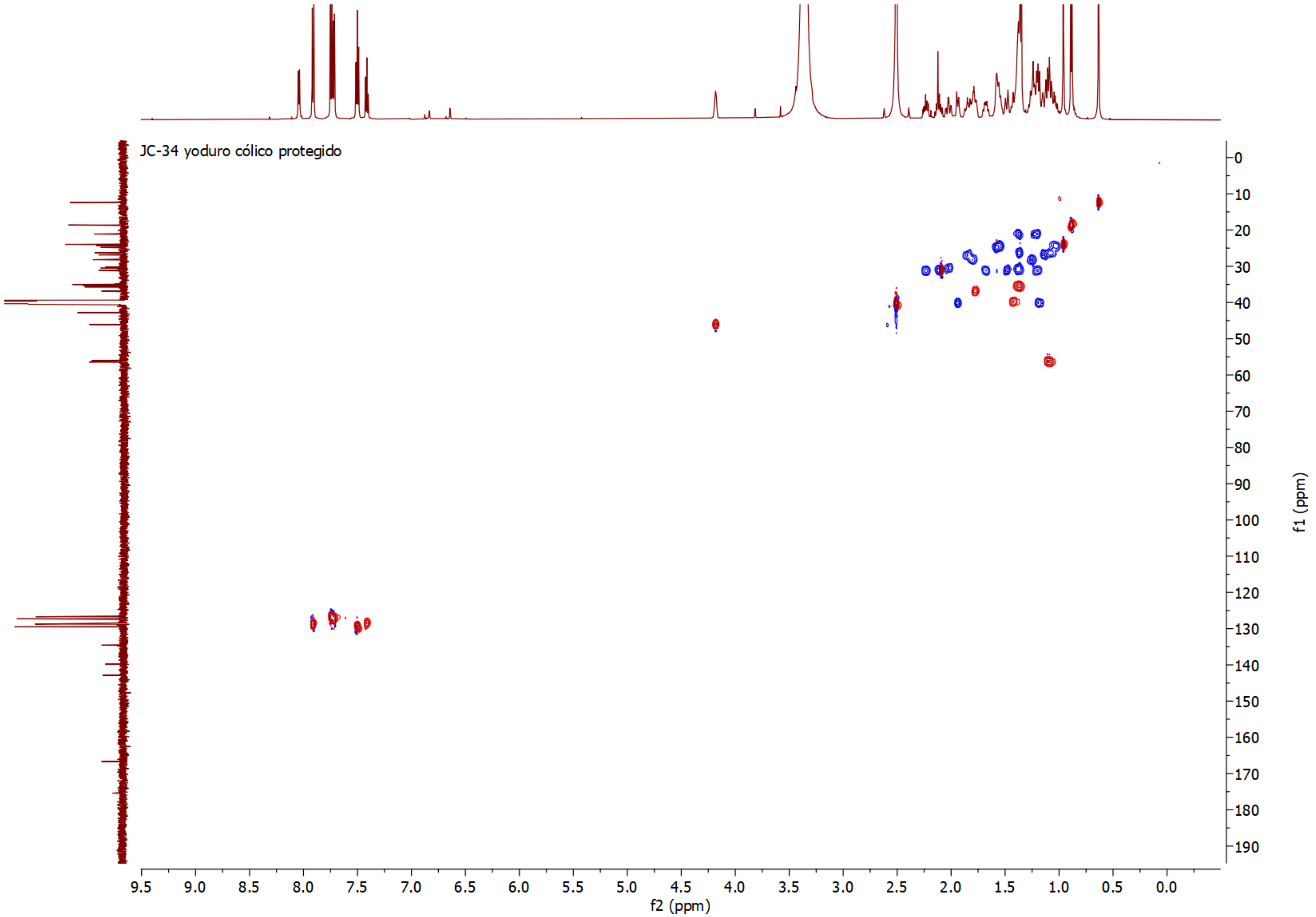

Figure S14. HSQC NMR spectrum of BIAC03L in DMSO-D6 (600 MHz).

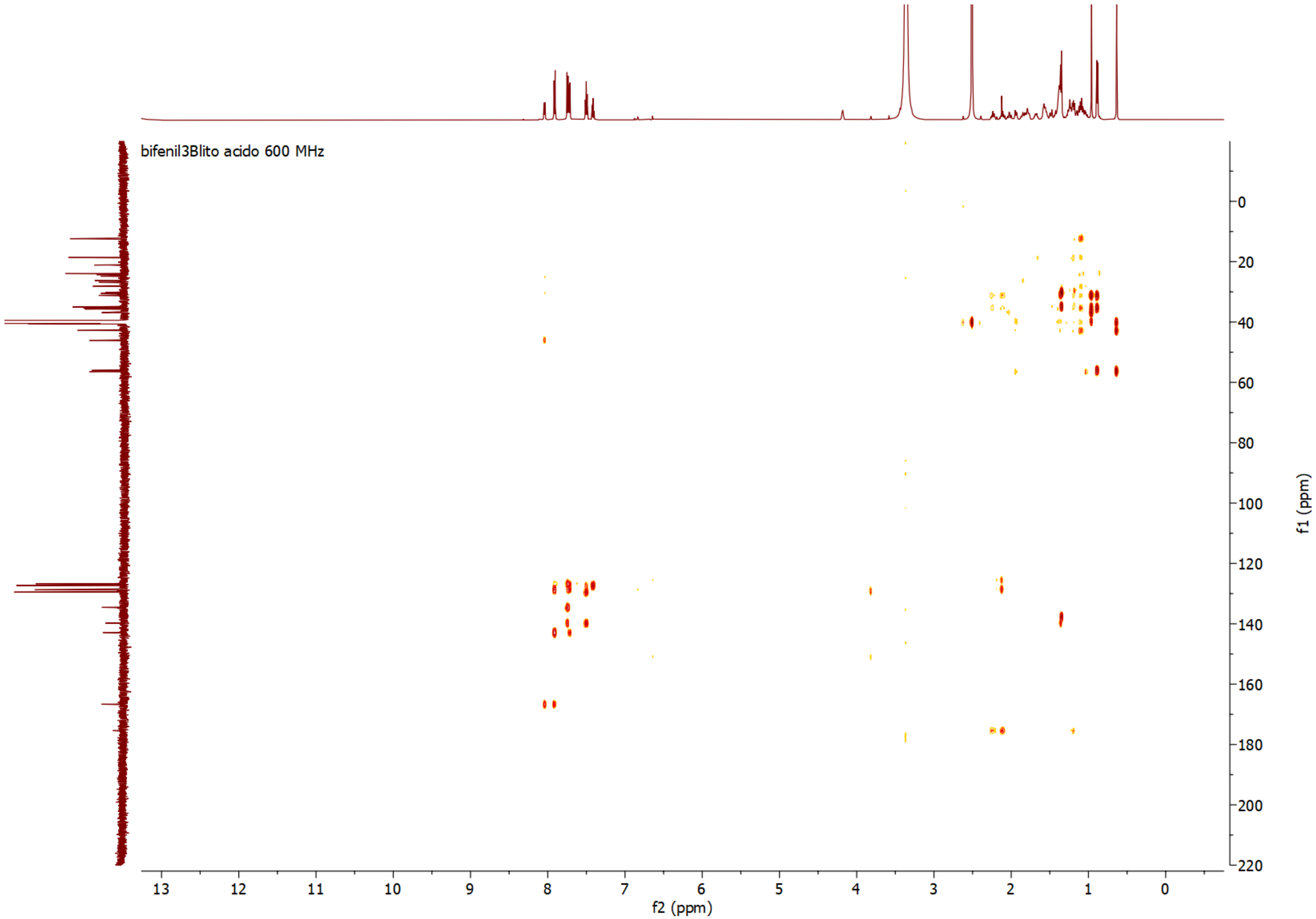

Figure S15. HMBC NMR spectrum of BIAC03L in DMSO-D6 (600 MHz).

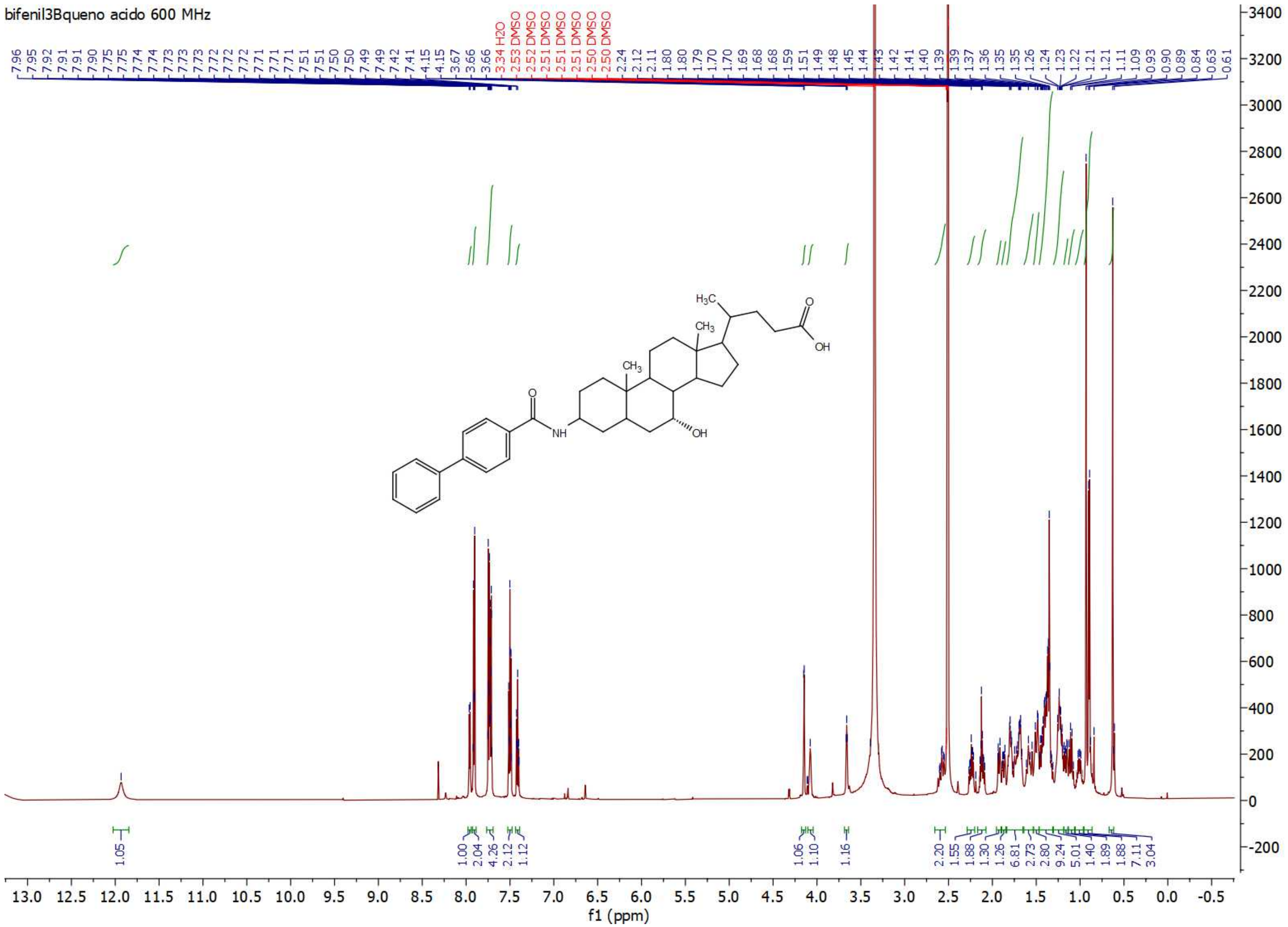

Figure S16. 1H-NMR spectrum of BIAC05Q in DMSO-D6 (600 MHz).

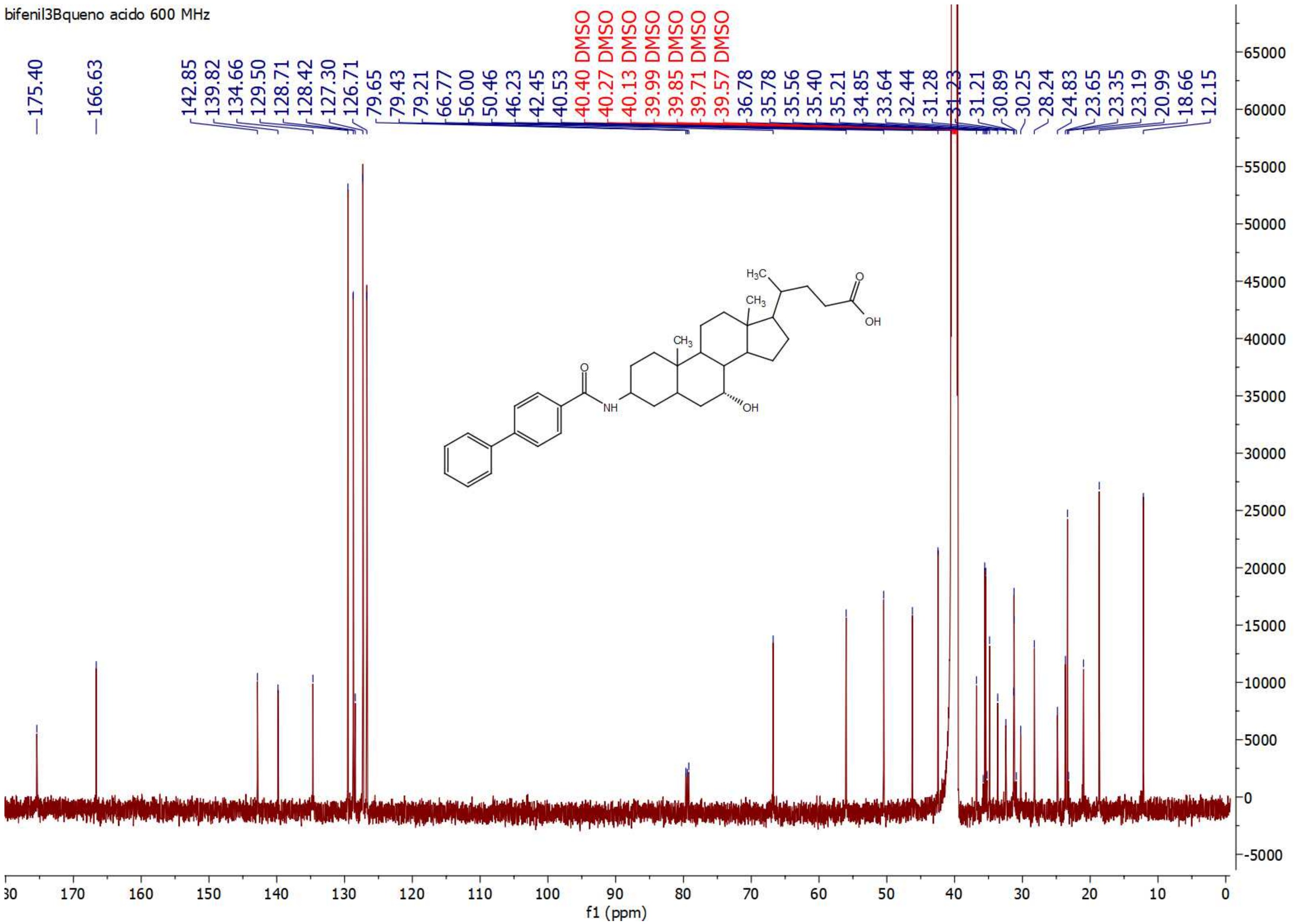

Figure S17. 13C-NMR spectrum of BIAC05Q in DMSO-D6 (600 MHz)

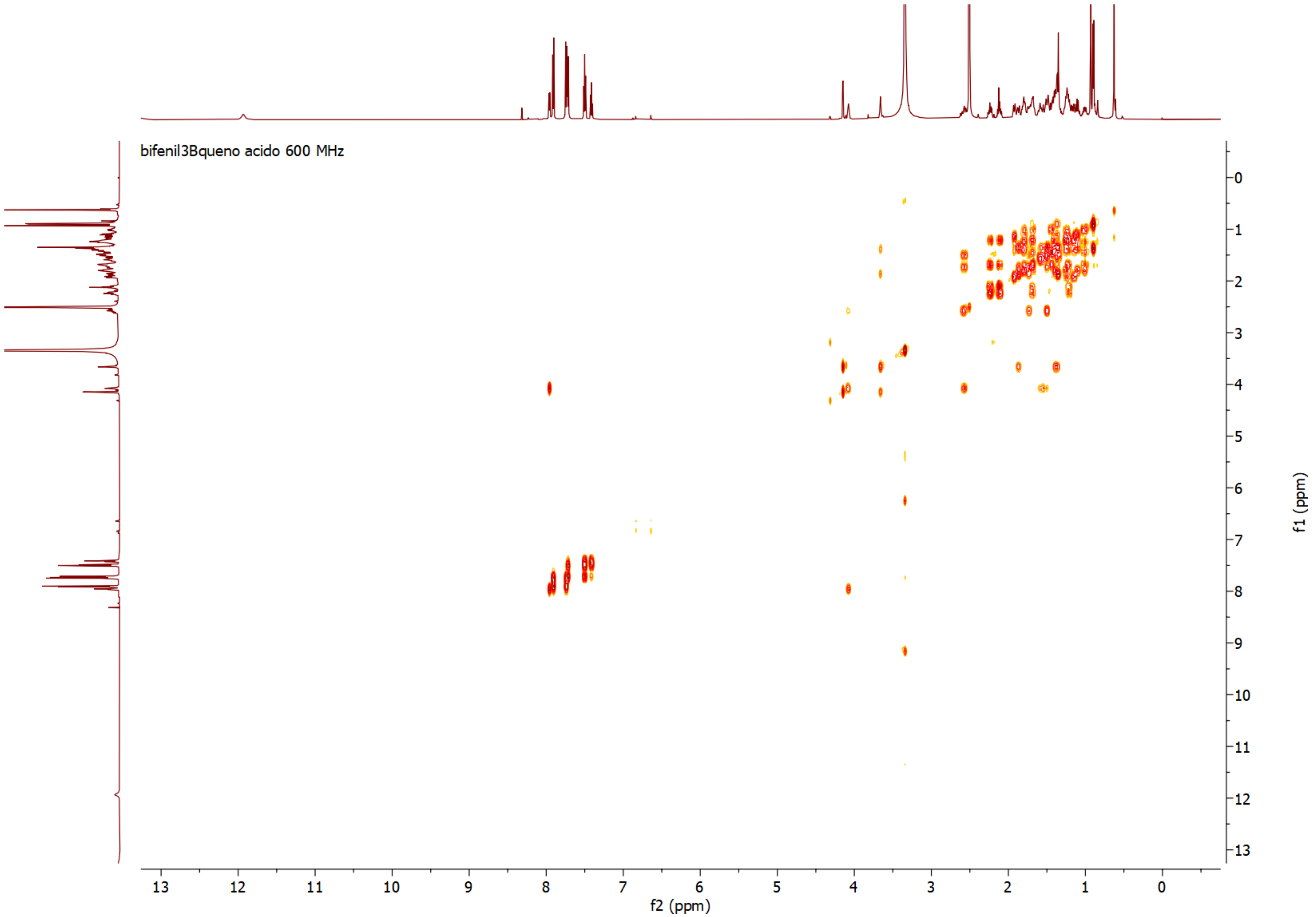

Figure S18. COSY-NMR spectrum of BIAC05Q in DMSO-D6 (600 MHz)

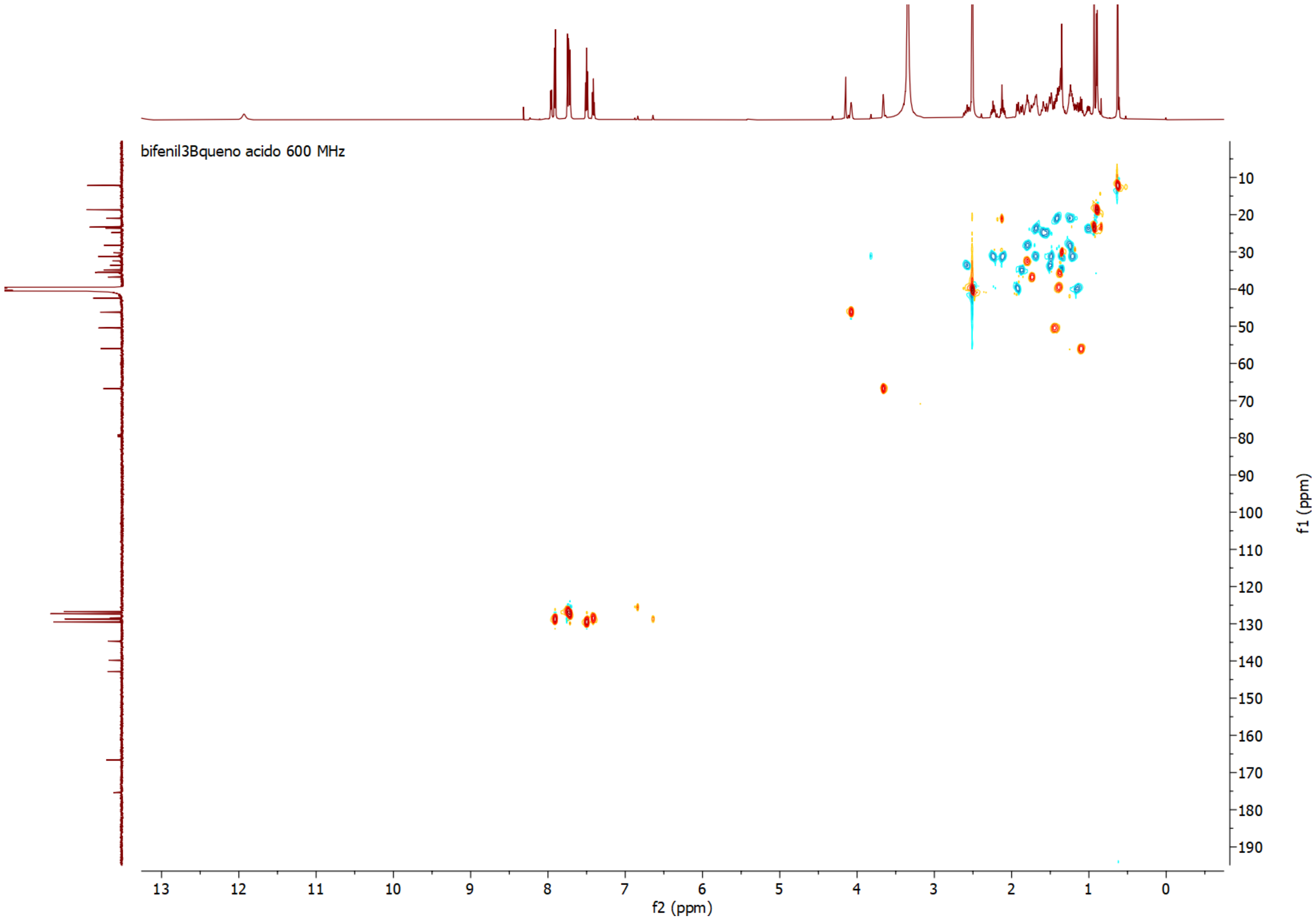

Figure S19. HSQC NMR spectrum of BIAC05Q in DMSO-D6 (600 MHz)

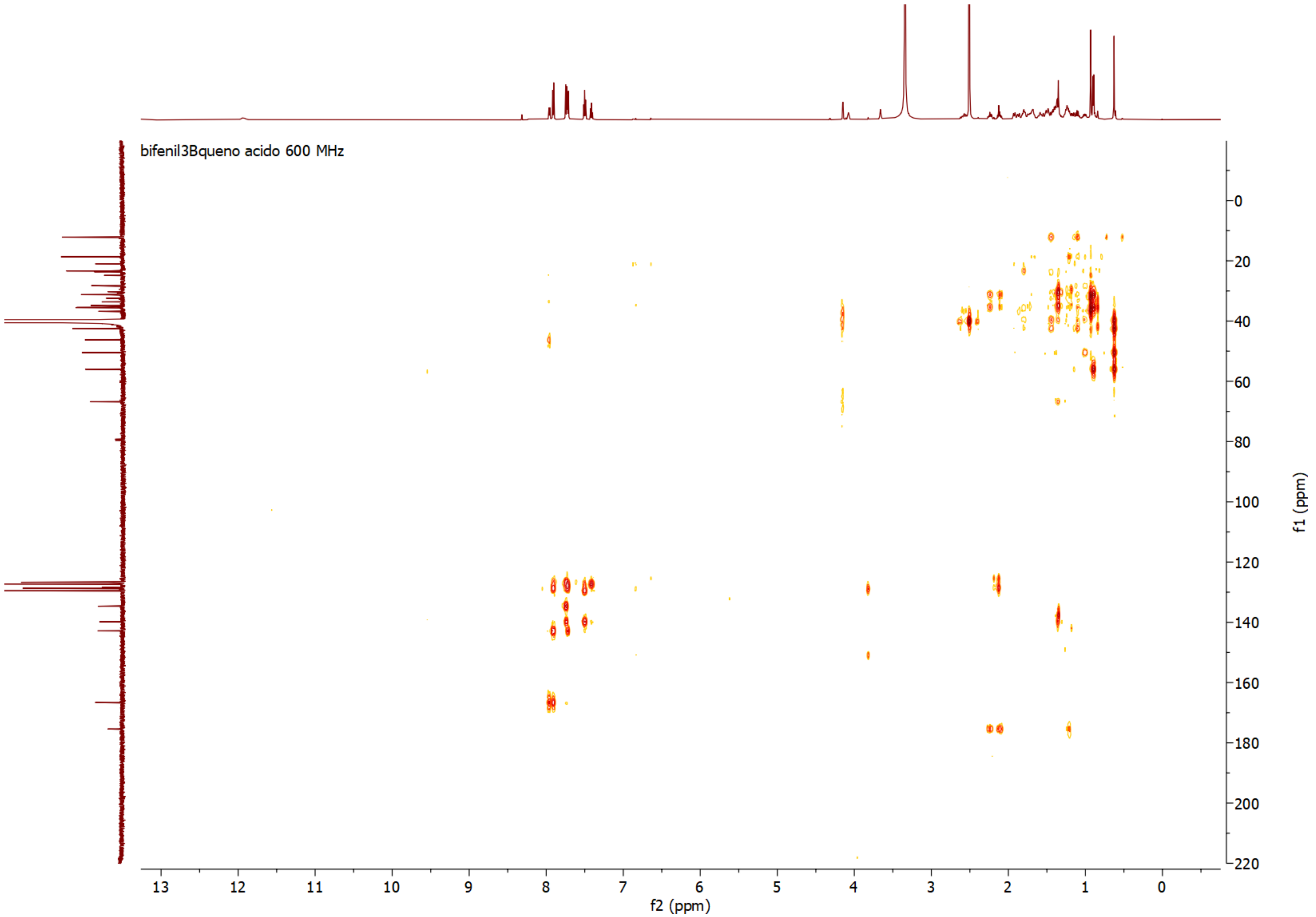

Figure S20. HMBC NMR spectrum of BIAC05Q in DMSO-D6 (600 MHz)

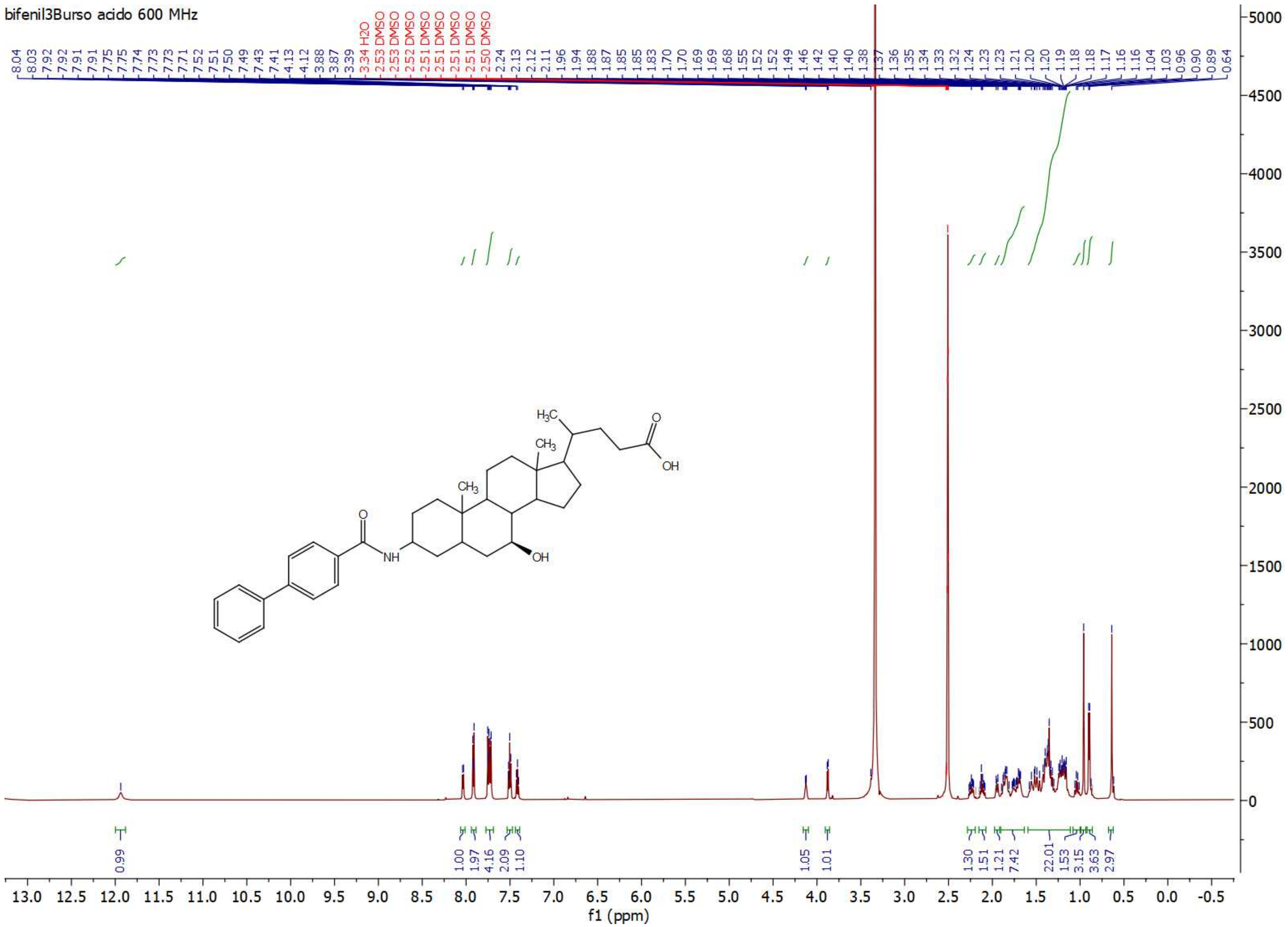

Figure S21. 1H-NMR spectrum of BIAC04U in DMSO-D6 (600 MHz)

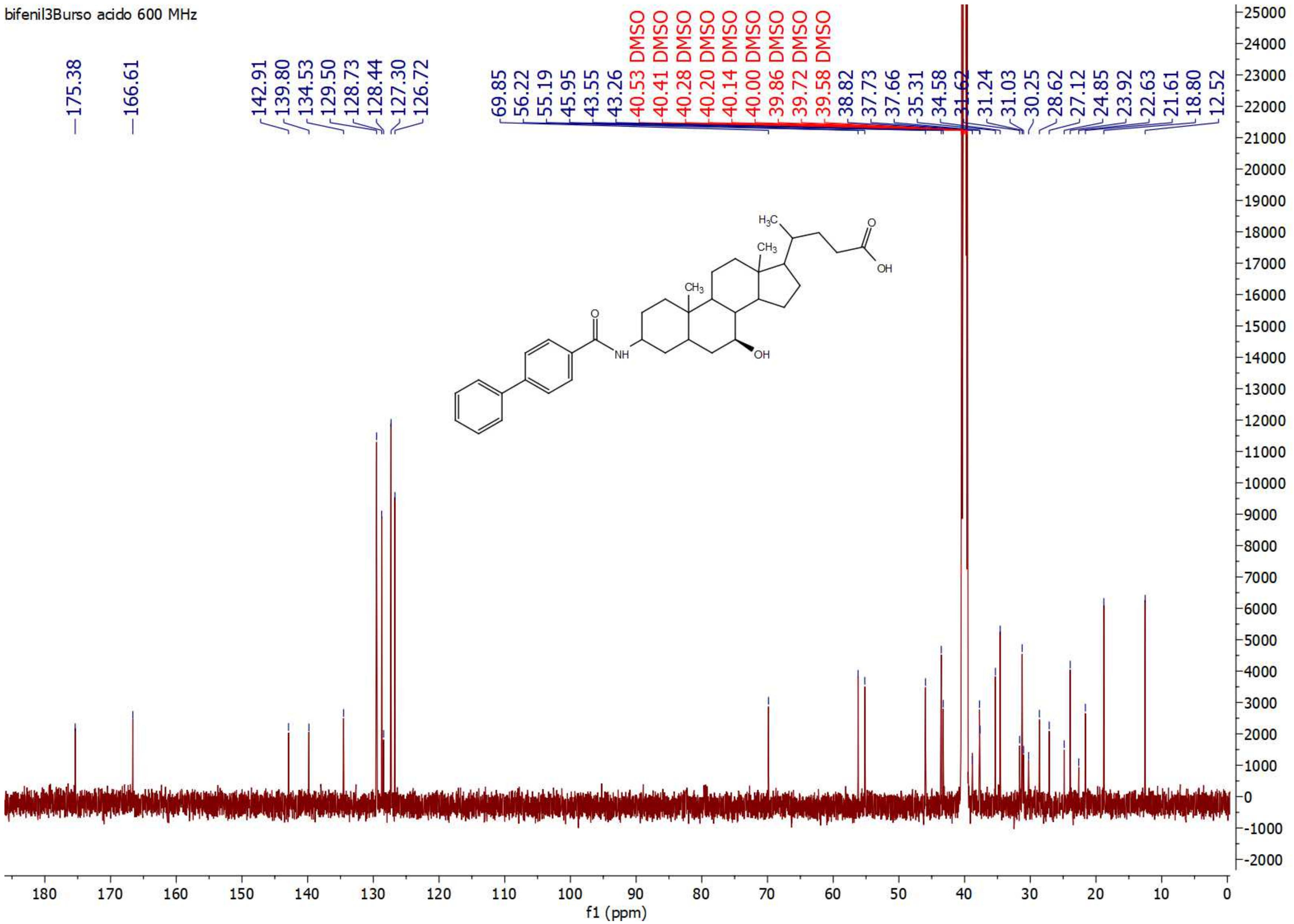

Figure S22. 13C-NMR spectrum of BIAC04U in DMSO-D6 (600 MHz)

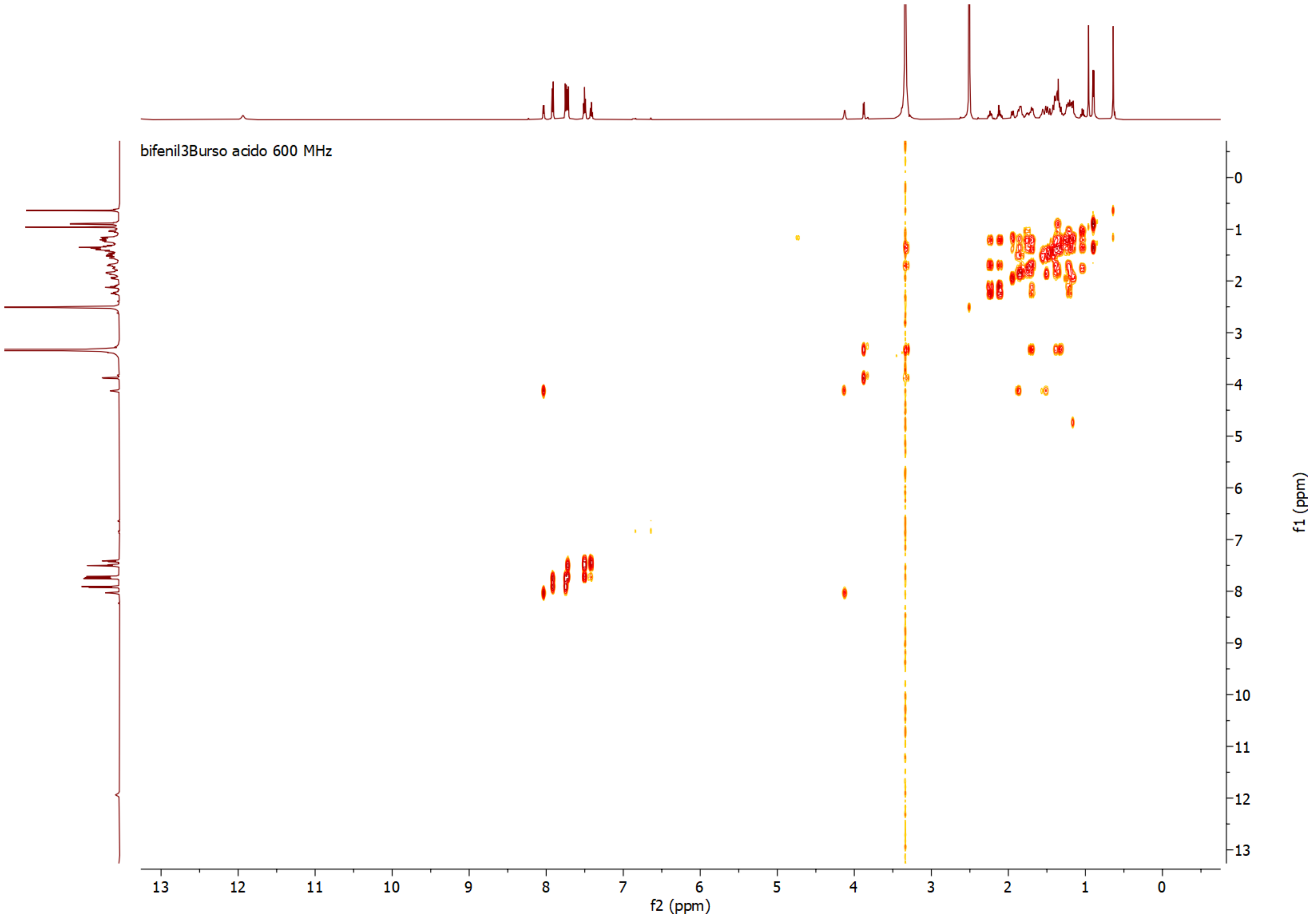

Figure S23. COSY NMR spectrum of BIAC04U in DMSO-D6 (600 MHz)

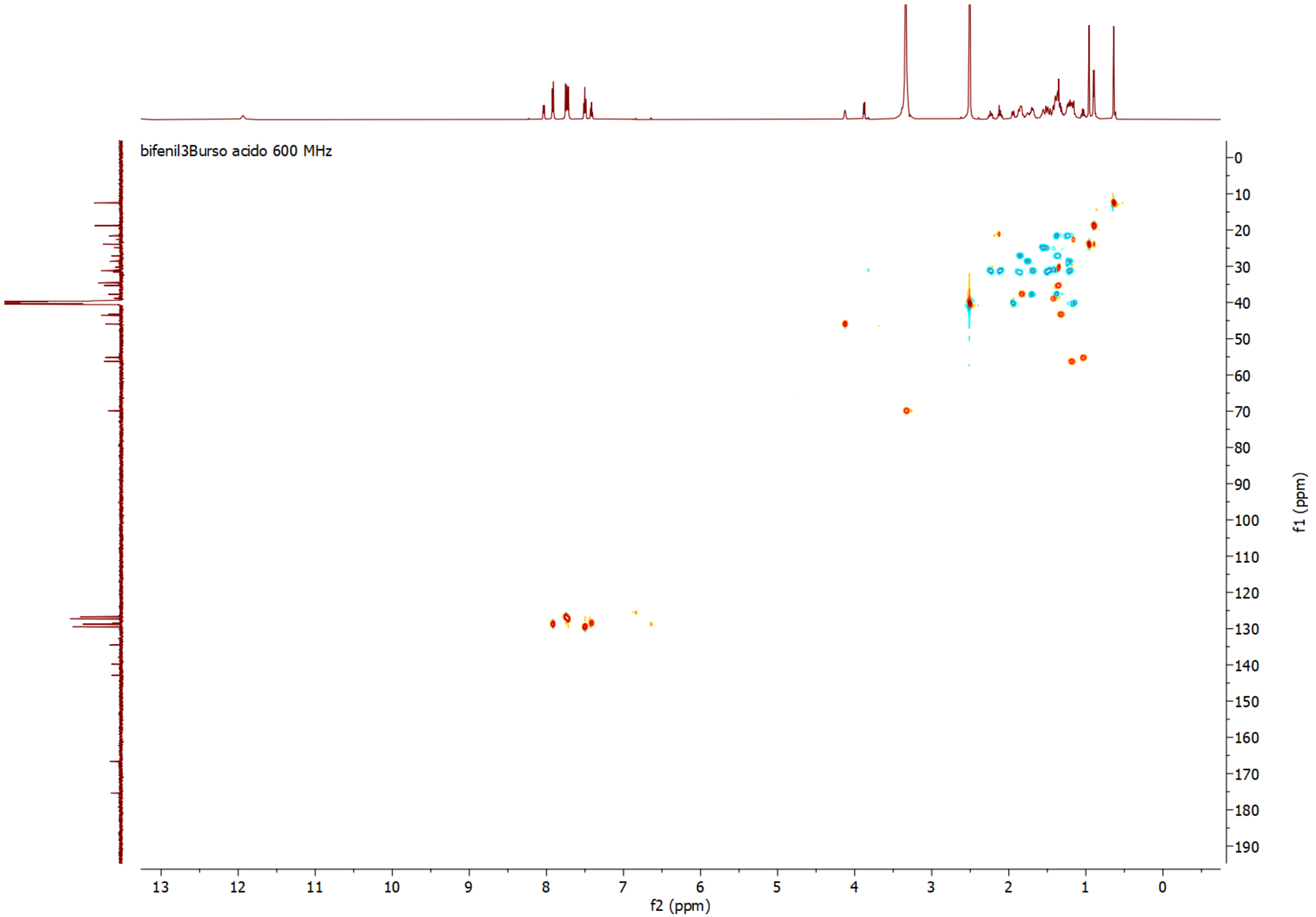

Figure S24. HSQC NMR spectrum of BIAC04U in DMSO-D6 (600 MHz)

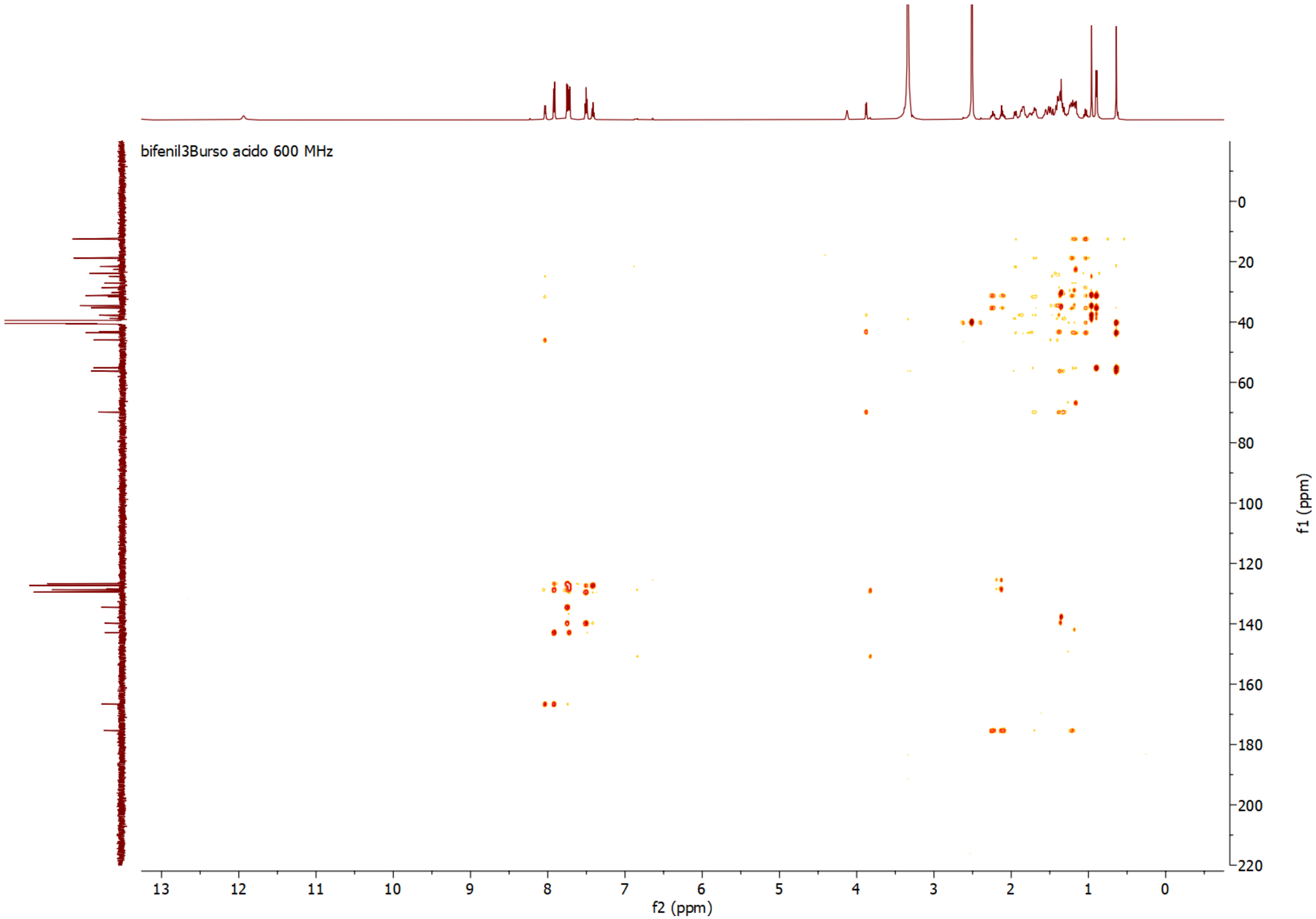

Figure S25. HMBC NMR spectrum of BIAC04U in DMSO-D6 (600 MHz)

ESI-Orbitrap-MS analysis

Samples were dissolved in methanol and measured in a Orbitrap Exploris 120, data were analyzed with Thermo Scientific Xcalibur version: 4.5.474.0

Compound	[M+H]	Calculated Mass	Current mass
BIAC01C	$C_{37}H_{50}O_5N^+$	588.36890	588.3686
BIAC02D	$C_{37}H_{50}O_4N^+$	572.37398	572.3738
BIAC03L	$C_{37}H_{50}O_3N^+$	556.37907	556.3781
BIAC04U	$C_{37}H_{50}O_4N^+$	572.37398	572.3727
BIAC05Q	$C_{37}H_{50}O_4N^+$	572.37398	572.3733

Table S5. HR-ESI-MS data of the synthesized compounds.

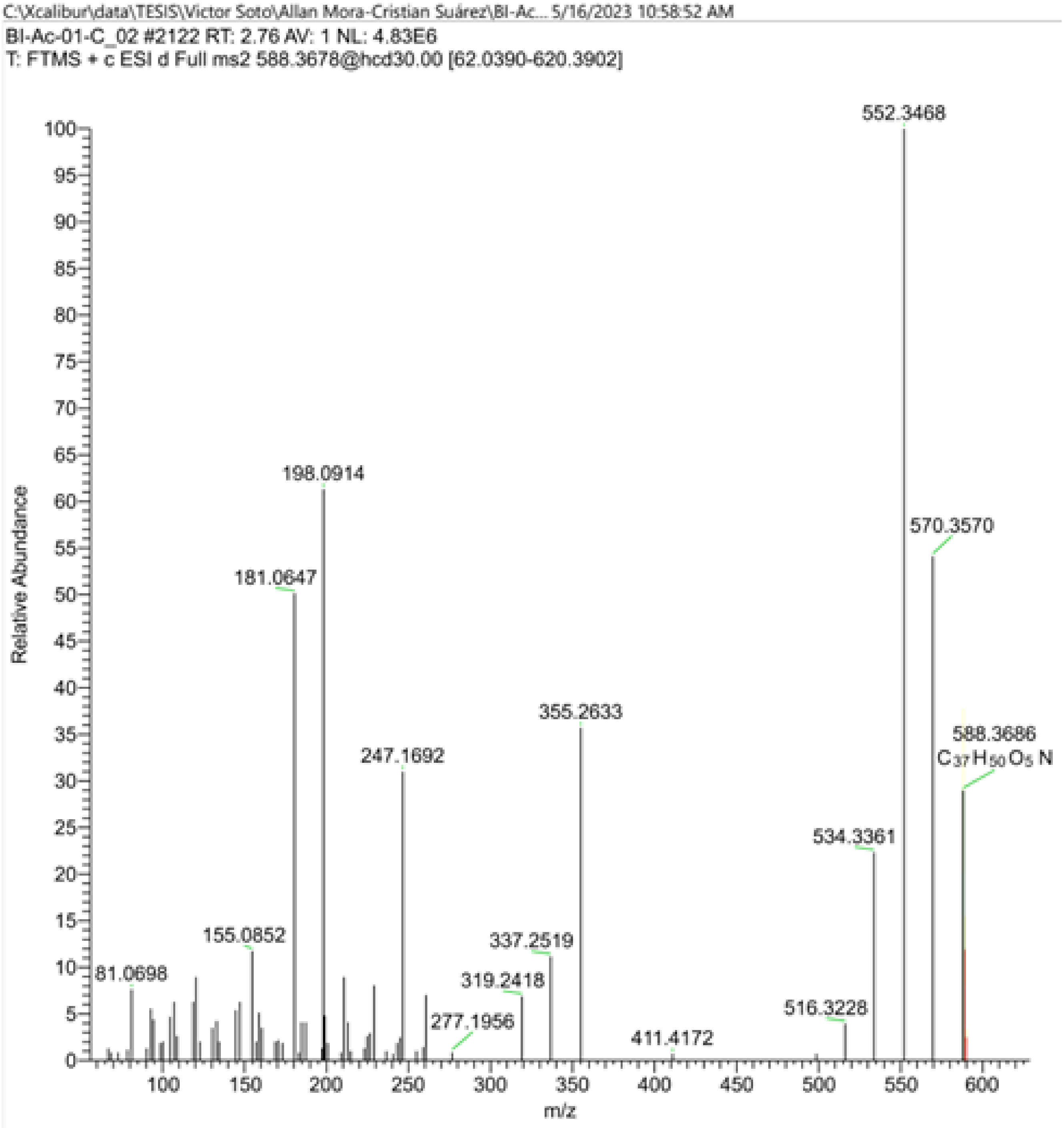

Peak Mass	Display Formula	Delta [ppm]	MSMS Matched Fragments
588.3686	$C_{37}H_{50}O_5N$	0.41	(Collection)

Figure S26. ESI-Orbitrap-MS of the Biphenyl Cholic acid derivative BIAC01C.

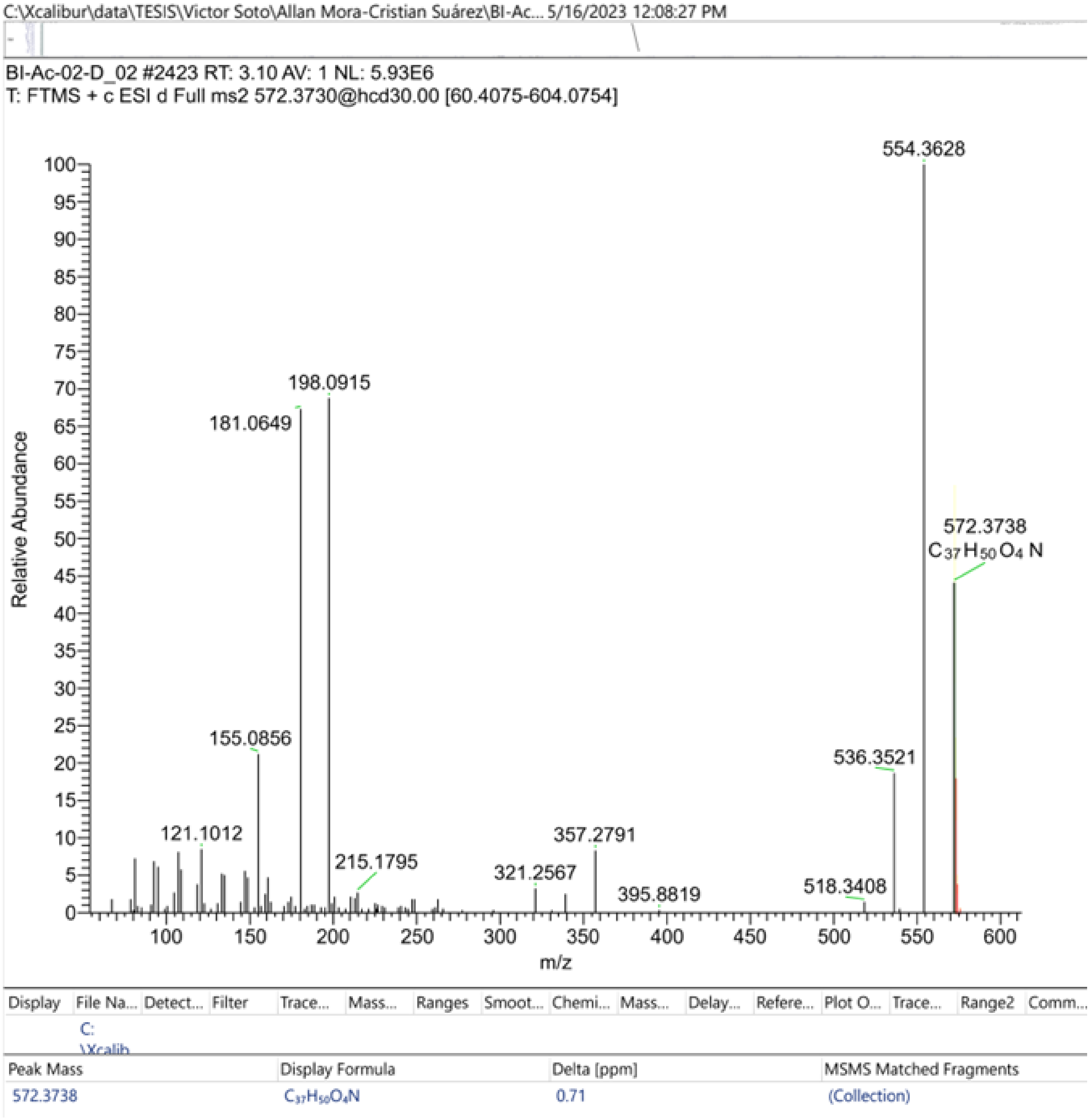

Figure S27. ESI-Orbitrap-MS of the Biphenyl Deoxycholic acid derivative BIAC02D.

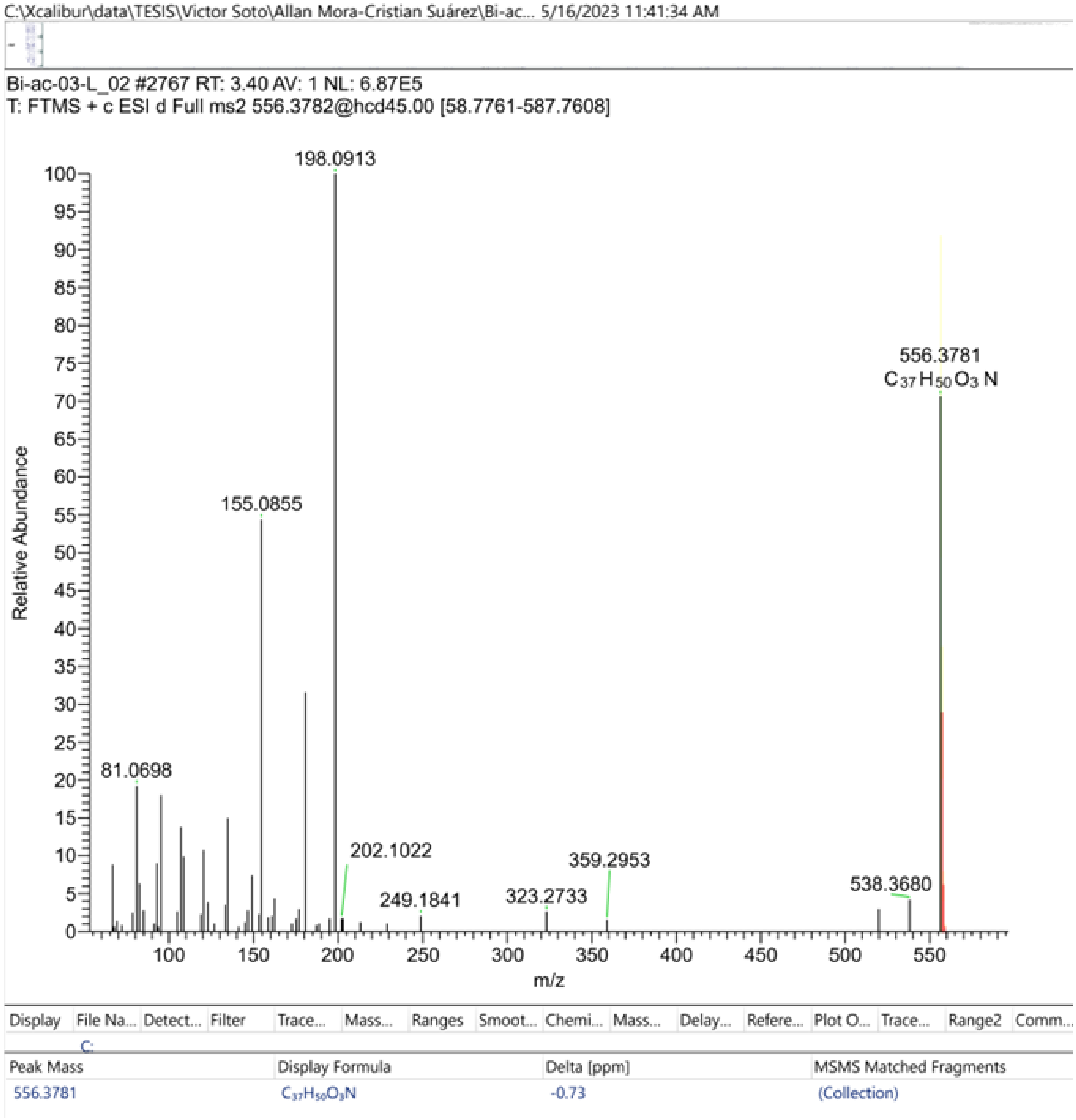

Figure S28. ESI-Orbitrap-MS of the Biphenyl Litocholic acid derivative BIAC03L.

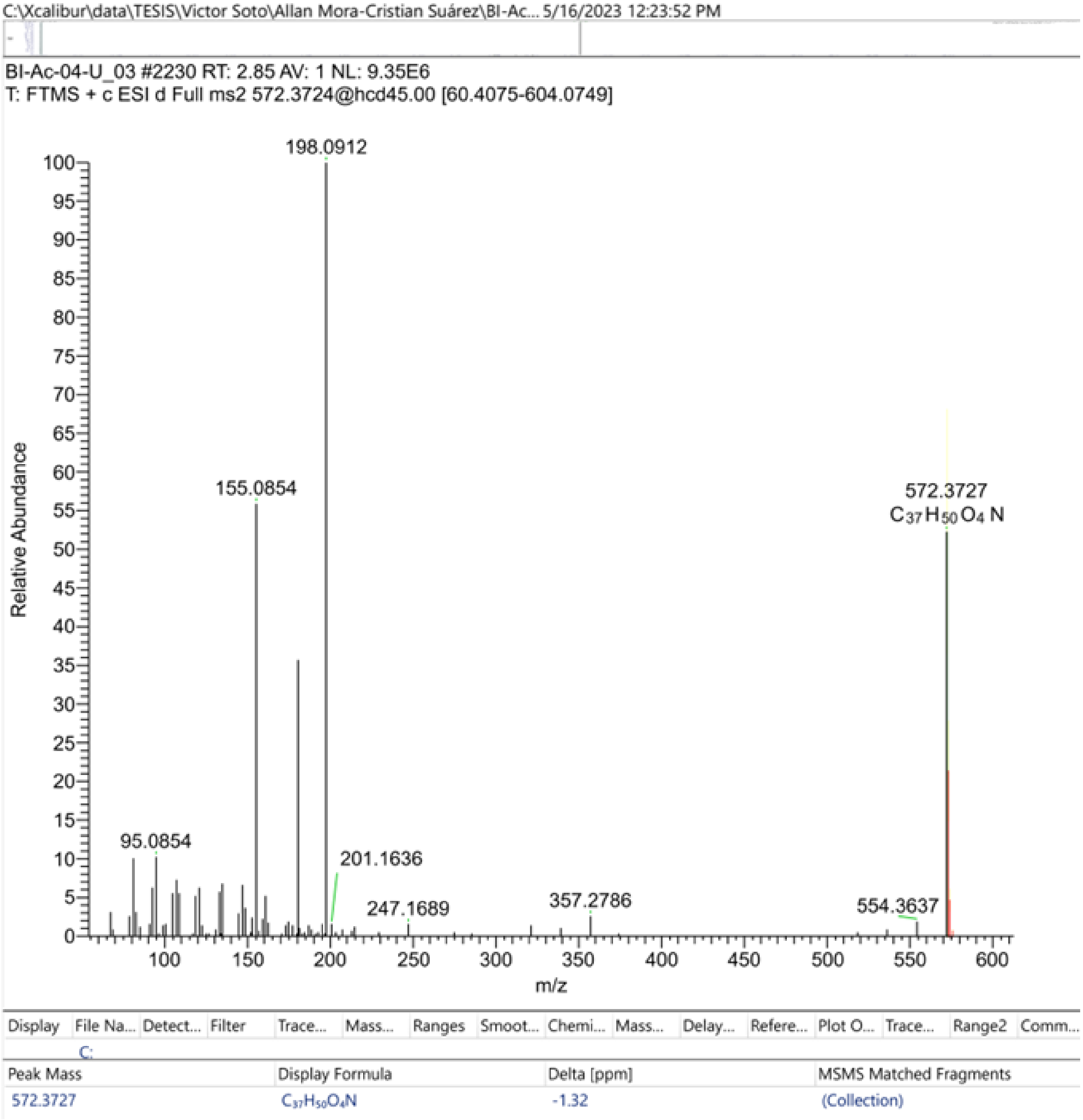

Figure S29. ESI-Orbitrap-MS of the Biphenyl Ursodeoxycholic acid derivative BIAC04U.

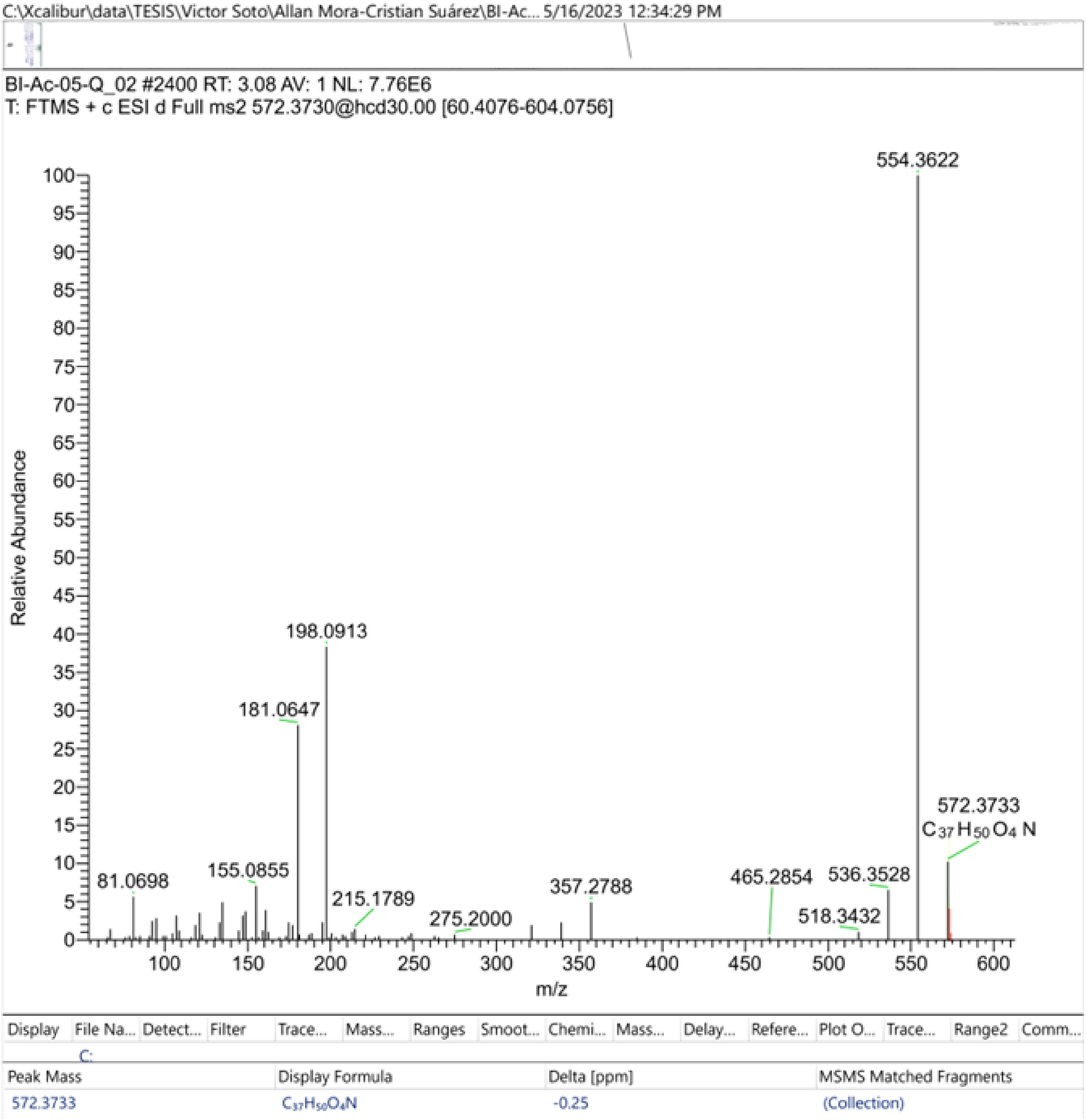

Figure S30. ESI-Orbitrap-MS of the Biphenyl Chenodeoxycholic acid derivative BIAC05Q.

3 Optical properties

The 4μm thick mesoporous TiO_2 films sensitized with the various dyenamo blue:coadsorber ratios were washed with a 0.5μL tetrabutylammonium hydroxide. This led to dye getting desorbed from the titania. 3mL of dichloromethane was added to the vial and the absorption spectra measured. J_{SC} values are reported from both direct J-V measurements under AM1.5G illumination and integrated from IPCE spectra (monochromatic illumination). The $\sim$ 10% difference between these values arises from different measurement conditions and is within typical margins for DSC characterization.

Device	J_{SC} (mA/cm^2)	V_{OC} (V)	FF	PCE (%)
Y123-CDCA	13.48	1.04	0.74	10.40
BIAC01C	13.12	1.03	0.75	10.08
BIAC02D	12.89	1.01	0.73	9.46
BIAC03L	12.29	1.03	0.74	9.37
BIAC04U	12.96	1.00	0.73	9.42
BIAC05Q	12.10	1.04	0.74	9.45

Table S6. Preliminary photovoltaic investigation on all five novel co-adsorbers using standard Y123 dye.

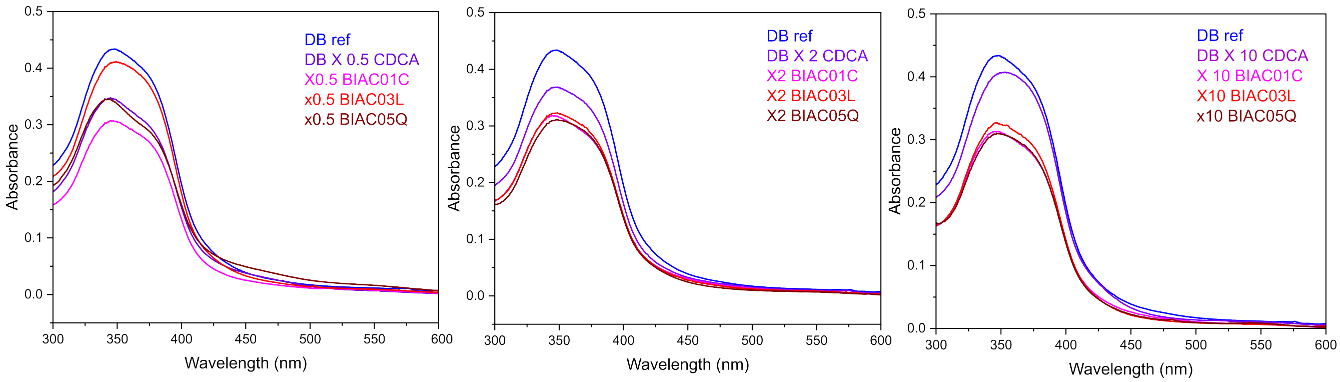

Figure S31. UV-Vis absorption spectra of the dye with different concentrations of the co-adsorbers (given in the legends) desorbed from the 4μm thick titania films.

4 Photovoltaic performance

**Dye:Co-sensitizer**	**Jsc (mA/cm2)**	**Voc (V)**	**FF**	**PCE (%)**
**0.075mM DB**	9.14 (7.56 ± 0.92)	0.74 (0.75 ± 0.01)	0.76 (0.74 ± 0.02)	5.17 (4.19 ± 0.64)
**1:10 DB:CDCA**	12 (11.68 ± 0.35)	0.74 (0.75 ± 0.01)	0.72 (0.72 ± 0.01)	6.4 (6.22 ± 0.16)
**1:10 DB:BIAC01C**	13.13 (12.53 ± 0.50)	0.76 (0.76 ± 0.01)	0.71 (0.72 ± 0.01)	7.11 (6.9 ± 0.16)
**1:10 DB:BIAC03L**	13.1 (12.95 ± 0.53)	0.77 (0.75 ± 0.01)	0.75 (0.73 ± 0.01)	7.56 (7.15 ± 0.35)
**1:10 DB:BIAC05Q**	13.1 (12.87 ± 0.41)	0.76 (0.76 ± 0.02)	0.73 (0.73 ± 0.01)	7.26 (7.09 ± 0.12)

Table S7. Photovoltaic performance with statistical analysis of the Dyenamo blue DSC devices based on the coadsorbers CDCA, BIAC01C, BIAC03L, BIAC05Q under AM1.5G sunlight

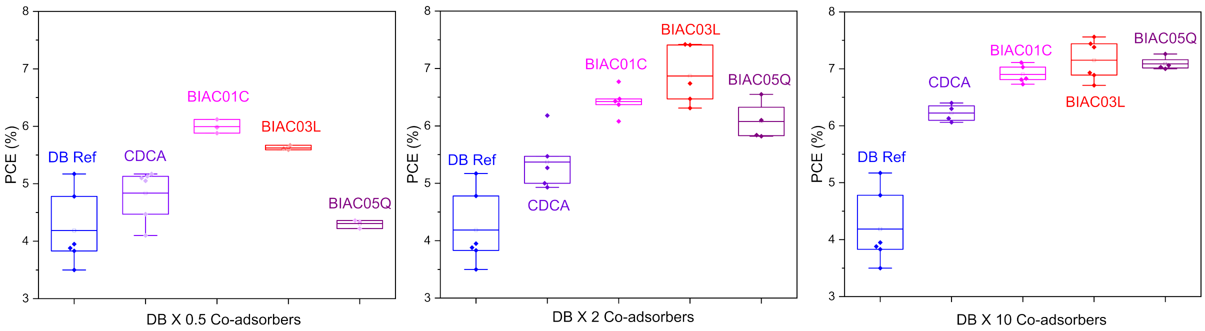

Figure S32. Statistical results showing the change in power conversion efficiencies of the devices upon increasing the concentration of the co-adsorbers from 0.5 times to 10 times the concentration of the dye dyenamo blue.

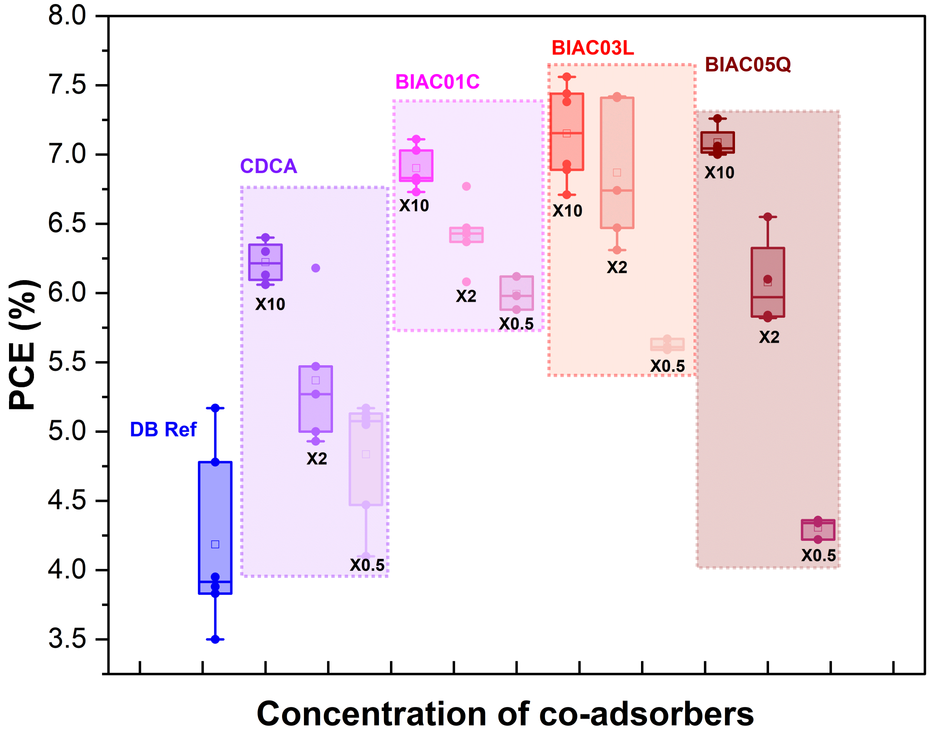

Figure S33. Statistical results showing the change in power conversion efficiencies as a function of concentration of the co-adsorbers

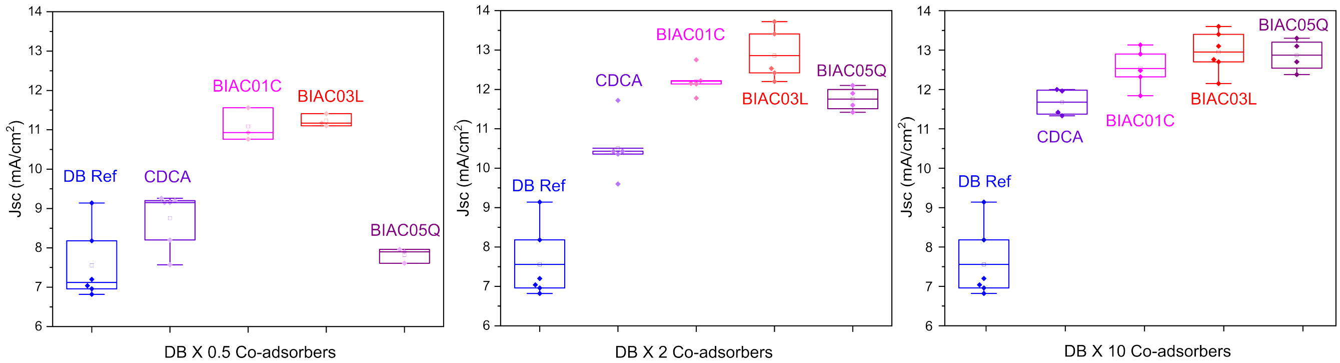

Figure S34. Statistical results showing the change in current densities of the devices upon increasing the concentration of the co-adsorbers from 0.5 times to 10 times the concentration of the dye dyenamo blue.

Dye:Co-sensitizer	**Jsc (mA/cm2)**	Voc (V)	FF	PCE (%)
0.075mM DB: D35	10.8<br>(10.68 ± 0.10)	0.78<br>(0.77 ± 0.01)	0.7<br>(0.69 ± 0.02)	5.87<br>(5.66 ± 0.23)
With CDCA	12.82<br>(12.63 ± 0.18)	0.79<br>(0.79 ± 0)	0.72<br>(0.72 ± 0.01)	7.19<br>(7.11 ± 0.09)
With BIAC01C	11.57<br>(11.45 ± 0.12)	0.75<br>(0.75 ± 0)	0.72<br>(0.72 ± 0)	6.24<br>(6.16 ± 0.07)
With BIAC03L	13.73<br>(13.59 ± 0.13)	0.77<br>(0.77 ± 0.01)	0.71<br>(0.71 ± 0.01)	7.37<br>(7.32 ± 0.06)
With BIAC05Q	12.8<br>(12.50 ± 0.30)	0.78<br>(0.77 ± 0.01)	0.73<br>(0.71 ± 0.03)	7.02<br>(6.9 ± 0.13)

Table S8. Photovoltaic performance of the Dyenamo blue:D35 co-sensitized DSC devices based on the coadsorbers CDCA, BIAC01C, BIAC03L, BIAC05Q under AM1.5G sunlight.

5 Photoluminescence spectrocopy

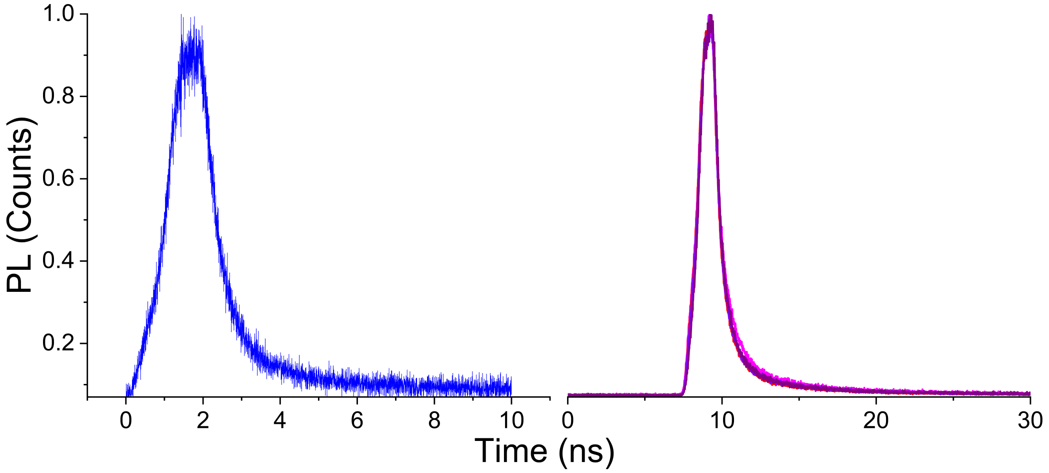

Figure S35. Time-resolved photoluminescence of DB dye and DB with the different coadsorbers on ZrO_2

	B1	tau1 (ns)	Rel 1 (%)	B2	tau2 (ps)	Rel 2 (%)
DB	358.775	0.61	100			
DB+ CDCA	3876.80	0.63	58.29	330.21	5.3	41.71
DB+ BIAC01C	2788.45	0.74	58.88	270.74	5.36	41.12
DB+ BIAC03L	8275.72	0.63	63.27	610	4.93	36.73
DB+ BIAC05Q	3511.72	0.63	61.32	277.48	5.06	38.68

Table S9. Fit parameters for photoluminescence decay lifetimes

6 Transient absorption spectroscopy

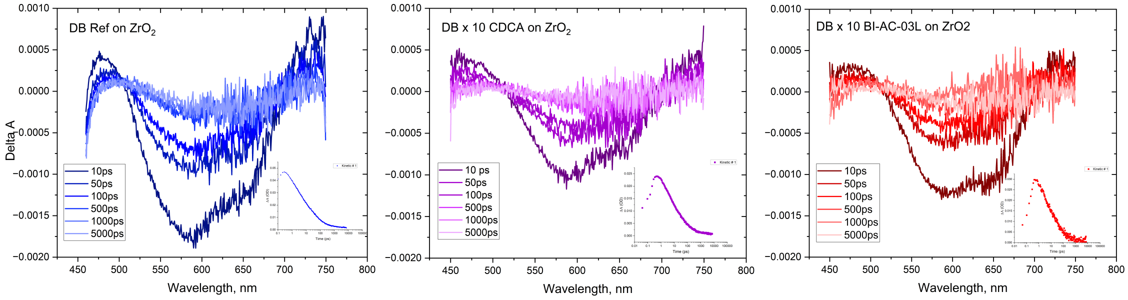

Figure S36. fs-TAS plots of DB dye and DB with the different coadsorbers on ZrO_2

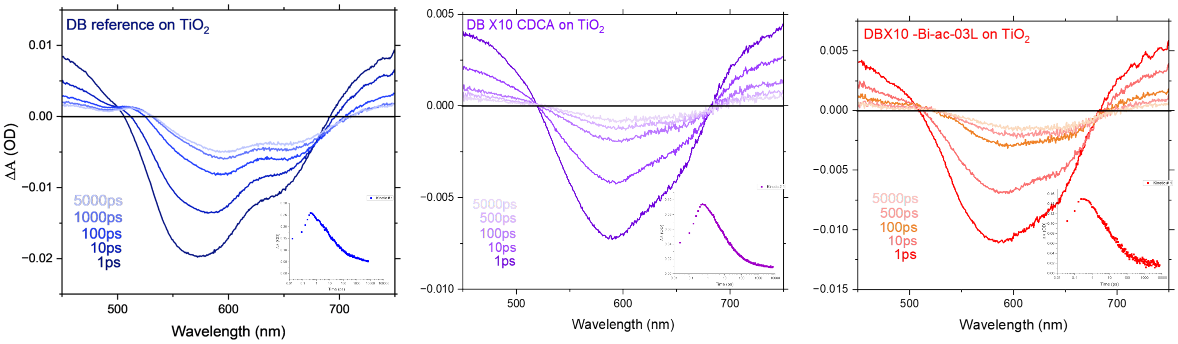

Figure S37. fs-TAS plots of DB dye and DB with the different coadsorbers on TiO_2

	On ZrO_2 rise (ps)	decay (ps)	On TiO_2 rise (ps)	decay (ps)
DB	3.4	77.85	6.4	140.5
DB+ CDCA	7.7	245.1	4.6	87.10
DB+ BIAC03L	7.04	188.9	4.6	90.48

Table S10. TA kinetic parameters for DB, DB+CDCA and DB+BIAC03L on ZrO_2 and TiO_2 observed at 710nm
