# Supplementary material for: Bile acid derivatives as novel co-adsorbents for enhanced performance of blue dye-sensitized solar cells
Source: Commun Chem. 2025 Mar 10;8:75. doi: 10.1038/s42004-025-01433-1 (PMC11894181; doi:10.1038/s42004-025-01433-1)
Supplement: Supplementary file 2 — Reporting Summary [file 42004_2025_1433_MOESM2_ESM.pdf]

nature portfolio

Corresponding author(s): Marina Freitag

Solar Cells Reporting Summary

Nature Portfolio wishes to improve the reproducibility of the work that we publish. This form is intended for publication with all accepted papers reporting the characterization of photovoltaic devices and provides structure for consistency and transparency in reporting. Some list items might not apply to an individual manuscript, but all fields must be completed for clarity.

For further information on Nature Research policies, including our data availability policy, see Authors & Referees.

Experimental design

Please check the following details are reported in the manuscript, and provide a brief description or explanation where applicable.

1. Dimensions

Item	Yes	No	Description / Explanation
Area of the tested solar cells	☒	☐	Main text, Methods section - Current-voltage measurements<br>*Explain why this information is not reported/not relevant.*
Method used to determine the device area	☒	☐	Main text, Methods section - Current-voltage measurements<br>*Explain why this information is not reported/not relevant.*

2. Current-voltage characterization

Item	Yes	No	Description / Explanation
Current density-voltage (J-V) plots in both forward and backward direction	☒	☐	Figure 2a, 3a
Voltage scan conditions	☒	☐	Main text, Methods section - Current-voltage measurements<br>*Explain why this information is not reported/not relevant.*
Test environment	☒	☐	Room temperature, air.<br>*Explain why this information is not reported/not relevant.*
Protocol for preconditioning of the device before its characterization	☐	☒	*Provide a description of the protocol.*<br>No preconditioning necessary.
Stability of the J-V characteristic	☐	☒	*Provide a description of the method used. The stability of the J-V characteristic can be verified with time evolution of the maximum power point or with the photocurrent at maximum power point; see ref. 5 for details.*<br>The forward and reverse J-V scans gave identical results- no unusual behaviour.

3. Hysteresis or any other unusual behaviour

Item	Yes	No	Description / Explanation
Description of the unusual behaviour observed during the characterization	☐	☒	*Provide a description of hysteresis or any other unusual behaviour observed during the characterization.*<br>No unusual behaviour observed.
Related experimental data	☐	☒	*Provide a description of the related experimental data.*<br>DSSCs do not usually show hysteresis behaviour.

4. Efficiency

Item	Yes	No	Description / Explanation
External quantum efficiency (EQE) or incident photons to current efficiency (IPCE)	☒	☐	Figure 2b and 3c<br>*Explain why this information is not reported/not relevant.*
A comparison between the integrated response under the standard reference spectrum and the response measure under the simulator	☒	☐	Figure 2b and 3c<br>*Explain why this information is not reported/not relevant.*

April 2023

For tandem solar cells, the bias illumination and bias voltage used for each subcell

- [ ] Yes
- [x] No

Provide a description of the measurement conditions.

We have not studied tandem systems.

5. Calibration

Light source and reference cell or sensor used for the characterization

- [x] Yes
- [ ] No

Main text, Methods section - Current-voltage measurements

Explain why this information is not reported/not relevant.

Confirmation that the reference cell was calibrated and certified

- [x] Yes
- [ ] No

Main text, Methods section - Current-voltage measurements

Explain why this information is not reported/not relevant.

Calculation of spectral mismatch between the reference cell and the devices under test

- [ ] Yes
- [x] No

Provide a value of the spectral mismatch and/or a description of how it has been taken into account in the measurements.

Before each set of measurements, the intensity was calibrated to 100 mW cm−2 using a certified silicon diode (Fraunhofer). Spectral mismatch calculations are not available.

6. Mask/aperture

Size of the mask/aperture used during testing

- [x] Yes
- [ ] No

Main text, Methods section - Current-voltage measurements

Explain why this information is not reported/not relevant.

Variation of the measured short-circuit current density with the mask/aperture area

- [ ] Yes
- [x] No

A circular mask was employed to confine the active solar cell area to 0.196 cm2

Explain why this information is not reported/not relevant.

7. Performance certification

Identity of the independent certification laboratory that confirmed the photovoltaic performance

- [ ] Yes
- [x] No

Identify the independent certification laboratory.

The focus of the paper is on understanding the role of the bile acid derivative co-adsorbents in increasing performance relative to the reference devices made in the same way. Certifying the absolute performance values is therefore not necessary in this case.

A copy of any certificate(s)

- [ ] Yes
- [x] No

Certificate copies should be provided in the Supplementary information. Please state the supplementary item number.

N/A

8. Statistics

Number of solar cells tested

- [x] Yes
- [ ] No

Main text, Methods section - Current-voltage measurements

Explain why this information is not reported/not relevant.

Statistical analysis of the device performance

- [x] Yes
- [ ] No

Main text, Figure 2d-e. 3b and SI, Figure S32, S33 and S34.

Explain why this information is not reported/not relevant.

9. Long-term stability analysis

Type of analysis, bias conditions and environmental conditions

- [ ] Yes
- [x] No

We make no claim about the long-term stability of the devices.

Explain why this information is not reported/not relevant.
